# Synthesis of novel photochromic pyrans via palladium-mediated reactions

**DOI:** 10.3762/bjoc.5.25

**Published:** 2009-05-27

**Authors:** Christoph Böttcher, Gehad Zeyat, Saleh A Ahmed, Elisabeth Irran, Thorben Cordes, Cord Elsner, Wolfgang Zinth, Karola Rueck-Braun

**Affiliations:** 1 Institut für Chemie, Technische Universität Berlin, Straße des 17. Juni 135, 10623 Berlin, Germany; 2 Permanent address: Chemistry Department, Faculty of Science, Assiut University, 71516 Assiut, Egypt; 3 Lehrstuhl für BioMolekulare Optik, Department für Physik, Ludwig-Maximilians-Universität München, Oettigenstraße 67, 80538 München, Germany

**Keywords:** benzopyrans, chromenes, naphthopyrans, palladium-mediated coupling reactions, photochromism

## Abstract

Photochromic pyrans for applications in material and life sciences were synthesized via palladium-mediated cyanation, carbonylation and Sonogashira cross-coupling starting from bromo-substituted naphthopyran **1** and benzopyrans **2a/b**. A novel photoswitchable benzopyran-based ω-amino acid **6** for Fmoc-based solid-phase peptide synthesis is presented. The photochromic behaviour of the 3-cyano-substituted benzopyran **5a** was investigated by time-resolved absorption spectroscopy in the picosecond time domain.

## Introduction

Interest in photoswitchable chromophores for the material and life sciences has increased dramatically over the past decade [1]. In the past, naphthopyrans were primarily commercialized as components for photochromic ophthalmic eyeware [2–5]. Nowadays, basic research in material sciences tries to implement photoswitchable pyrans and related chromophores as functional parts in organo-electronic devices, e.g. as logic gates [1]. In the life sciences, functionalized azobenzenes [6], hemithioindigos [7–8] and fulgimides [9–10] among other classes of photoswitches, have already proven to be valuable tools for photo-controlling the structure and function of complex biomolecules [6–12]. In this regard also a broad range of photoswitchable functionalized pyrans and spiropyrans seem to be promising candidates [13–14], but they are in addition interesting optical probes for fluorescence-based imaging technologies [15–16].

**Scheme 1:**
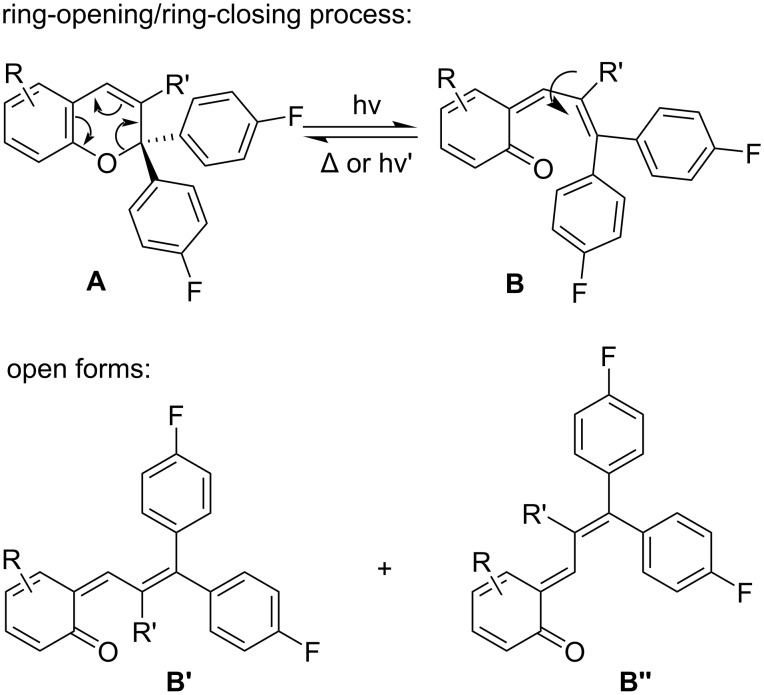
Photochromism of 2*H*-chromenes.

UV light-illumination of naphtho- and benzopyrans (closed forms), also referred to as chromenes, leads to the formation of interconverting photoisomers of the open merocyanine-type (quinoidal, zwitterionic and hybrid forms and their stereoisomers), depending upon the substitution pattern of the parent pyrans and the conditions employed (Scheme 1). Typically the thermal reversion of the open forms is very fast [17–20].

Quite recently, the formation of allene intermediates at low temperatures was also reported in the literature. They originate from open merocyanine isomers and are formed via a 1,5-hydrogen shift reaction [21–22]. However, these allene intermediates can be avoided by the replacement of hydrogen in the immediate vicinity of the two aryl residues (3-position of 2*H*-1-benzopyrans and 2-position of 3*H*-naphtho[2,1-*b*]pyrans). Thereby, the photochromism of benzo- and naphthopyrans is expected to be simplified for biological applications and also in material sciences.

**Scheme 2:**
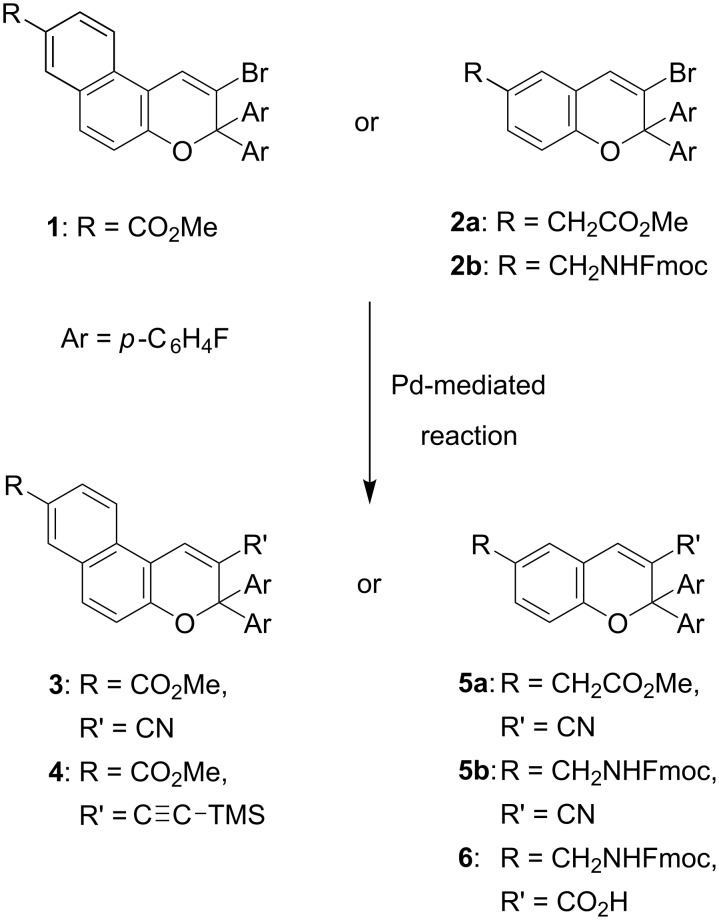
Synthesis of functionalized pyrans from 2-bromo-3*H*-naphtho[2,1-*b*]pyrans and 3-bromo-2*H*-1-benzopyrans by palladium-mediated transformations.

The design of the novel functionalized naphtho- and benzopyrans **3** and **5** presented herein is based on the substitution of the hydrogen in the neighbourhood of the two aryl residues by a cyano group, and additionally on fluoro-substituted aryl moieties (Scheme 2).

This substitution pattern permits detailed nitrile and fluorine spectroscopic probing of the photochromism in solution and on surfaces over a broad temperature range. Furthermore, the closed form of the benzopyran-based ω-amino acid **6** (Scheme 2) might be an ideal photoswitchable β-turn mimetic. Optical stimulation generates a flexible C3–C4 single bond within the open merocyanine forms in place of the former double bond of the pyran ring (Scheme 1), resulting in a significant structural alteration of the β-turn scaffold.

Herein we describe the synthesis of functionalized photochromic pyrans by palladium-catalyzed transformations of the bromo-substituted naphthopyran **1** and the benzopyrans **2a/b** (Scheme 2). A variety of transformations for the synthesis of 4-substituted benzopyrans of pharmacological interest (potassium channel openers), starting from triflate and bromo precursors, is well documented in the literature [23–24]. In comparison, reported transformations for 3-bromo-benzopyrans and bromo-substituted naphthopyrans are still rare. One reason could be the well documented anionic cleavage of 3-bromo-substituted benzopyrans upon metal halogen exchange, resulting in the formation of allenes and subsequent rearrangement reactions [25].

Since nitriles are important intermediates in the synthesis of carboxylic acids and derivatives, and for the introduction of aminomethyl substituents, we started our studies with palladium-catalyzed cyanation reactions. The studies were then extended towards a Heck carbonylation in the presence of an Fmoc protecting group for the direct synthesis of ω-amino acid **6**. Also, a Sonogashira reaction was conducted towards the synthesis of naphthopyran precursor **4**, which is suitable for attachment to tripodal linker systems for the immobilization on surfaces [26].

## Results and Discussion

**Scheme 3:**
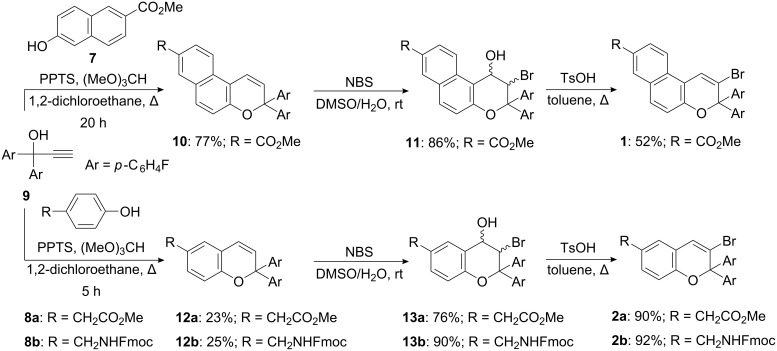
Synthesis of the 2-bromo-3*H*-naphtho[2,1-*b*]pyran **1** and the 3-bromo-2*H*-1-benzopyrans **2a/b**.

The starting materials, propargyl alcohol **9** and the phenol **8b**, were prepared essentially by following literature procedures (Scheme 3). Synthesis of the pyran precursors **10** and **12a/b** was accomplished by using PPTS and trimethyl orthoformate in DCE in a standard protocol developed by Carreira [27] for the Claisen-type rearrangement of propargyl aryl ethers in situ formed. All attempts to optimize the yield of **12a/b** failed, because of the decomposition reactions observed over prolonged reaction times and the difficulties encountered during the separation of the byproducts by flash chromatography. Treatment of **10** and **12a/b** with NBS in wet DMSO furnished the bromohydrin intermediates **11** and **13a/b** in good to excellent yields. The bromo-substituted fused pyrans **1** and **2a/b** were obtained in high yields by using TsOH in toluene for the subsequent elimination of water [25].

Since arenenitriles are important for the fine chemical industry, a variety of methods have been developed recently for the efficient cyanation of aryl halides under mild conditions. By applying electron-rich phosphanes and either Zn(CN)_2_ or K_4_[Fe(CN)_6_], the transformation of chlorides to nitriles was successfully achieved under fairly mild conditions. However, protocols for vinyl systems are still rare.

When using Zn(CN)_2_ (0.6 equiv) in the presence of Pd(dba)_2_/[(*t*-Bu)_3_PH]BF_4_ with Zn (0.05 equiv) as additive in wet NMP (0.2% water, 0.03 M) at 45 °C, no conversion of the bromo-substituted naphthopyran **1** was observed after 17 h. Additional experiments showed that the conversion of **1** could be improved by activation of the zinc powder using TMSCl or iodine, whereas bromine was less efficient (Table 1).

**Table 1:** Optimization of palladium catalyzed cyanation reactions with Zn(CN)_2_.

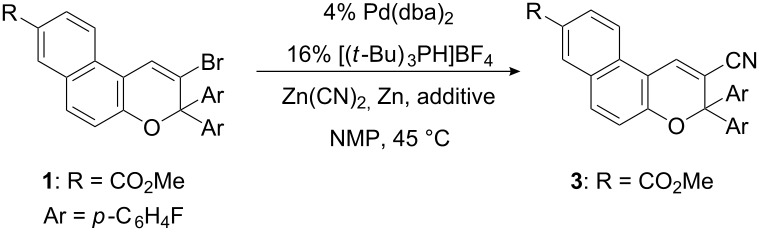						
entry	concn (M)	Zn(CN)_2_ (equiv)	additive (equiv)	time (h)	conversion^a^ (%)	yield^a,b,c^ (%)

1	0.03	0.6	0.2 Br_2_	18	7	5
2	0.03	0.6	0.2 TMSCl	19	29	23
3	0.03	0.6	0.2 I_2_	17	56	54
4	0.50^d^	1.1	0.2 I_2_	41	100	97^e^

^a^Determined by RP-HPLC at 210 nm. ^b^HPLC-based yield. ^c^2–5% of a byproduct, that was not further characterized, were observed by HPLC. ^d^8% [(*t*-Bu)_3_PH]BF_4_ were used. ^e^Isolated yield after purification by flash-chromatography: 83%.

When using iodine, 56% conversion was determined by RP-HPLC (Table 1, entry 3). Under related reaction conditions with a selection of other ligands (PEPPSI, dppf, dpppe) the transformation could not be further enhanced.

For complete conversion the concentration of the reaction mixture was raised from 0.03 M to 0.5 M, 1.1 equiv Zn(CN)_2_ was applied and the reaction time was doubled. Thereby, compound **3** could be isolated in 83% yield after work up and flash chromatography. In other solvents, the reaction either did not proceed (toluene, THF) or was less efficient (DMF).

**Scheme 4:**
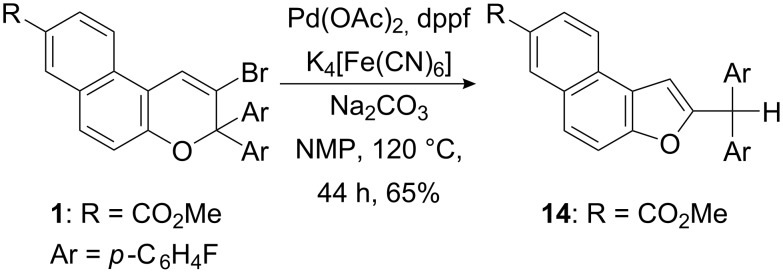
Ring contraction observed during the cyanation approach towards the synthesis of **3**.

The cyanation of vinyl bromide **1** was also investigated by using K_4_[Fe(CN)_6_] in the presence of Pd(OAc)/dppf and Na_2_CO_3_ in NMP at 120 °C for 44 h (Scheme 4) [28]. However, only the rearranged and hydrodehalogenated benzofuran **14** was isolated in 65% yield after flash chromatography.

**Scheme 5:**
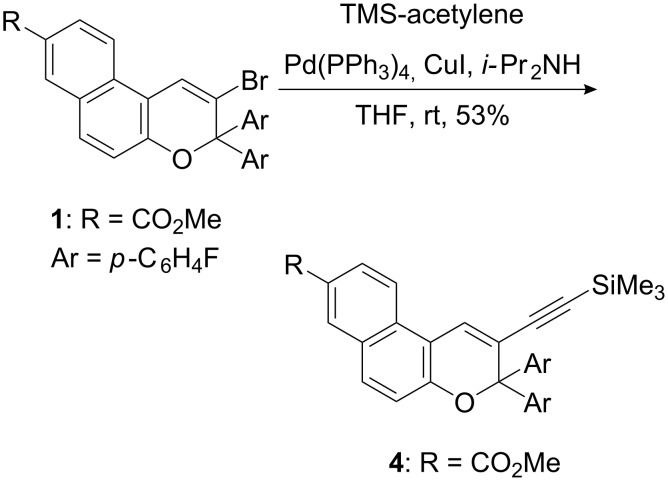
Palladium-catalyzed Sonogashira-coupling of 2-bromo-3*H*-naphtho[2,1-*b*]pyran **1**.

Formation of the benzofuran can be rationalized by cleavage of the benzopyran ring and a subsequent rearrangement reaction [29–30]. However, by using standard Sonogashira conditions in THF, (trimethylsilyl)acetylene was reacted with **1** for 45 h to yield **4** in 53% after work-up and purification by flash chromatography (Scheme 5).

**Scheme 6:**
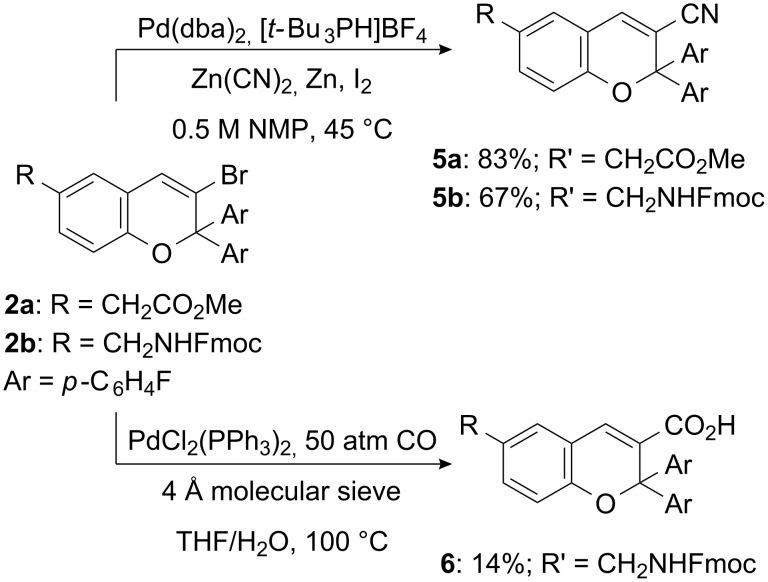
Palladium-catalyzed cyanation and carbonylation of 3-bromo-2*H*-1-benzopyrans **2a/b**.

The Zn(CN)_2_-based cyanation protocol was also successfully utilized in the transformation of the sterically demanding vinyl bromides **2a/b**, affording **5a** and **5b** in good to excellent yields (Scheme 6).

Starting from the bromo-substituted pyran **2b** a final carbonylation step (Scheme 6) would directly complete the synthesis of an Fmoc-protected ω-amino acid. Though many protocols for Heck carbonylations are known, only a few report neutral reaction conditions, necessary here because of the sensitivity of the Fmoc protecting group. However, in a procedure published by Urata et al. [31] the use of molecular sieve instead of base was successful. The reaction outcome was dependent on the molecular sieves type, since the pore diameter must correspond to the ion size of the halides used. We were pleased to isolate 200 mg of the ω-amino acid **6** starting from a 2.3 mmol reaction scale of **2b**, when using water in THF for 72 h at 100 °C and 50 atm CO in the presence of PdCl_2_(PPh_3_)_2_ and 4 Å molecular sieves. The low yield of 14% could be attributed to decomposition of starting material and product under the harsh reaction conditions. All attempts to use MeOH in THF under similar conditions failed to produce detectable amounts of the desired methyl ester.

### Photochemistry of benzopyrans: Compound **5a**

**Figure 1:**
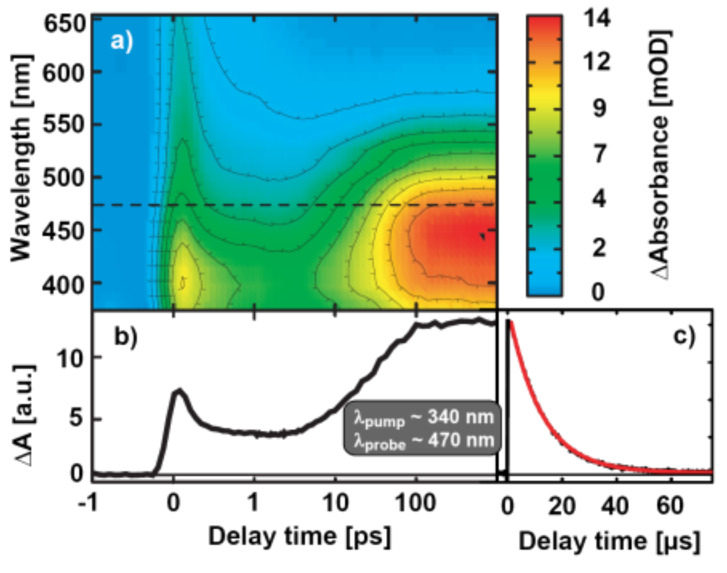
Data from time-resolved measurements of compound **5a**. a) and b): Results from fs-pump-probe-spectroscopy. Please note the linear time-scale from −1 ps to 1 ps and the logarithmic scale thereafter. a) Two-dimensional overview plot of the transient absorbance changes in false colour coding. a.u. = arbitrary units. b) Temporal behaviour of the absorbance changes at a detection wavelength of 470 nm. c) Results from laser-flash photolysis. The transient was recorded at 470 nm (black) and is shown together with a single-exponential fit (red) yielding a decay time of 12 μs.

Photophysical properties of the novel pyrans are exemplified with compound **5a**: in stationary measurements no indications for the formation of the open form, i.e. appearance of a visible absorption band after UV-illumination, were found. Therefore spectroscopic techniques with much higher time-resolution were applied to record the reaction dynamics of **5a** and to search for its “ring-opened” forms. The photoreaction was initiated by short light-pulses at 340 nm. The corresponding transient absorption changes (375–650 nm) as a function of the delay-time between pump and probe are shown in Figure 1a. An inspection of the data at early delay-times (0.2 ps) reveals a broad and unstructured induced absorption in the complete spectral range. This feature vanishes on fast timescales < 1 ps and leads to a weak absorption increase in the 400 nm range. On the time scale of 30 ps a pronounced absorption band in the visible appears, which peaks at ~460 nm.

This spectral and temporal behaviour is already known from other naphtho- and benzopyrans as well as for spiropyrans [20,32]. The initial transients are assigned to the first excited singlet state [32]. Global fitting of the data set reveals a bi-exponential decay of the excited state absorption with the time constants 0.15 ps and 0.9 ps. The low absorption and the spectral position (~400 nm) of the band observed subsequently point to the formation of ring-opened molecules with a distorted non-planar structure with these time constants. The coloured “open” forms appear on a longer time-scale (*τ* = 31 ps) related with the rise of an intense band centered at ~460 nm (Figure 1a/b). The spectral signature of this band allows an assignment to the open forms with a more planar structure due to the sp^2^-hybridization at the formerly sp^3^-hybridized carbon carrying the aryl residues. The π-system now extends over a considerable fraction of the molecule.

The time constant for the formation of the open form (31 ps) is slower than the reaction dynamics recorded for other benzopyrans [20,32]. The slow reaction speed may be related to the cyano substituent at the 3-position in the benzopyran moiety (compare Scheme 2). A detailed publication comparing substances with and without substituents in this position is in preparation.

The results given in Figure 1a/b clearly demonstrate that the ring-opening reaction proceeds on a picosecond timescale. Since the stationary measurements do not reveal a change of absorption under light exposure, the open intermediate should be short-lived. The laser-flash experiments of Figure 1c show the kinetics of the back reaction. The band of the open form (e.g. Figure 1a, *t*_D_ = 1000 ps) vanishes mono-exponentially with a time-constant of 12 μs (Figure 1c). Similar lifetimes were found for related systems [20].

The photochemistry of the investigated benzopyran **5a** may be summarized as follows: as known from the literature [32], a very short-lived excited state is observed after photoexcitation into the S_1_-band, which decays on a time-scale < 1 ps. The molecule has reached the open form with some distortion. Planarization and the resulting built-up of strong absorption in the visible occurs on the time-scale of several ten picoseconds. The open form lives only for a limited time: the visible absorption band centered at 460 nm is found to disappear on the timescale of ten microseconds.

## Conclusion

In conclusion, we have synthesized novel photochromic naphtho- and benzopyrans for applications in the material and life sciences. A mild and efficient palladium-catalyzed cyanation reaction of bromo-substituted naphtho- and benzopyrans using Zn(CN)_2_ has been developed. Also a Sonogashira cross-coupling furnishing naphthopyran **4** and a palladium-catalyzed carbonylation yielding ω-amino acid **6** has been carried out. Detailed studies on nitrile transformations and alternative carbonylation routes in the presence of other protecting groups are currently under way in our laboratory. Time-resolved photophysical spectroscopy of benzopyran **5a** could also demonstrate a significant influence of the substitution in 3-position on the reaction dynamics of the ring-opening process.

## Supporting Information

Full Experimental procedures, characterization data and copies of spectra for **1**–**6**, **8b** and **9**–**14**. Description of the time-resolved measurements and X-ray analysis of compounds **1**, **3** and **5a**.

File 1 Experimental Part
