# Supplementary material for: Synthesis of novel photochromic pyrans via palladium-mediated reactions
Source: Beilstein J Org Chem. 2009 May 27;5:25. doi: 10.3762/bjoc.5.25 (PMC2707076; doi:10.3762/bjoc.5.25)
Supplement: File 1 — Experimental Part [file Beilstein_J_Org_Chem-05-25-s001.doc]

**Supporting Information**

**Synthesis of Novel Photochromic Pyrans via Palladium-Mediated Reactions**

**Experimental Part**

C. Böttcher^1^, G. Zeyat^1^, S. A. Ahmed^1,3^, E. Irran^1^, T. Cordes^2^, C. Elsner^2^, W. Zinth^2^ and Karola Rueck-Braun^*,1^

*^1^Technische Universität Berlin, Institut für Chemie,*

*Straße des 17. Juni 135, D-10623 Berlin, Germany*

*^2^Lehrstuhl für BioMolekulare Optik, Department für Physik,*

*Ludwig-Maximilians-Universität München,*

*Oettigenstraße 67, 80538 München, Germany*

*^2^Permanent address: Chemistry Department, Faculty of Science, Assiut University,*

*71516 Assiut, Egypt*

*karola.rueck-braun@tu-berlin.de*

**Table of contents**

**Part I Synthesis and characterization**

General notes S 5 – S 6

Four-step Synthesis of Compound **8b** – Experimental Procedures for the

Preparation of the Precursors

4-(Allyloxy)benzonitrile[[1]](#footnote-2) S 7

4-(Allyloxy)benzylamineError: Reference source not found S 8

(9*H*-Fluoren-9-yl)methyl [4-(allyloxy)benzyl]carbamate S 9 – S 10

(9*H*-Fluoren-9-yl)methyl (4-hydroxybenzyl)carbamate (**8b**)Error: Reference source not found S 10 – S 11

1,1-Bis(4-fluorophenyl)prop-2-yn-1-ol (**9**)Error: Reference source not found S 11 – S 12

Experimental Procedures for the Preparation of Functionalized Benzo- and Naphthopyrans

3,3-Bis(4-fluorophenyl)-8-(methoxycarbonyl)-3*H*-naphtho[2,1-*b*]pyran (**10**) S 12 – S 13

2-Bromo-3,3-bis(4-fluorophenyl)-8-(methoxycarbonyl)-2,3-dihydro-

1*H-*naphtho[2,1-*b*]pyran-1-ol (**11**) S 13 – S 14

2-Bromo-3,3-bis(4-fluorophenyl)-8-(methoxycarbonyl)-

3*H*-naphtho[2,1-*b*]pyran (**1**) S 15 – S 16

2,2-Bis(4-fluorophenyl)-6-[(methoxycarbonyl)methyl]-2*H*-1-benzopyran (**12a**) S 16 – S 17

2,2-Bis(4-fluorophenyl)-6-({[(9*H*-fluoren-9-yl)methoxycarbonyl]amino}methyl)-

2*H*-1-benzopyran (**12b**) S 17 – S 18

3-Bromo-2,2-bis(4-fluorophenyl)-6-[(methoxycarbonyl)methyl]-

3,4-dihydro-2*H*-1-benzopyran-4-ol (**13a**) S 18 – S 19

3-Bromo-6-({[(9*H*-fluoren-9-yl)methoxycarbonyl]amino}methyl)-

2,2-bis(4-fluorophenyl)-3,4-dihydro-2*H*-1-benzopyran-4-ol (**13b**) S 20 – S 21

3-Bromo-2,2-bis(4-fluorophenyl)-6-[(methoxycarbonyl)methyl]-

2*H*-1-benzopyran (**2a**) S 21 – S 22

3-Bromo-2,2-bis(4-fluorophenyl)-6-({[(9*H*-fluoren-9-yl)methoxycarbonyl]-

amino}methyl)-2*H*-1-benzopyran (**2b**) S 22 – S 23

2-Cyano-3,3-bis(4-fluorophenyl)-8-(methoxycarbonyl)-3*H*-naphtho[2,1-*b*]pyran (**3**) S 24 – S 25

3-Cyano-2,2-bis(4-fluorophenyl)-6-[(methoxycarbonyl)methyl]-

2*H*-1-benzopyran (**5a**) S 26 – S 27

3-Cyano-2,2-bis(4-fluorophenyl)-6-({[(9*H*-fluoren-9-yl)methoxycarbonyl]amino}-

methyl-2*H*-1-benzopyran (**5b**) S 27 – S 29

2-[Bis(4-fluorophenyl)methyl]-7-(methoxycarbonyl)naphtho[2,1-*b*]furan (**14**) S 29 – S 30

3,3-Bis(4-fluorophenyl)-8-(methoxycarbonyl)-2-[(trimethylsilyl)ethinyl]-

3*H*-naphtho[2,1-*b*]pyran (**4**) S 31 – S 32

2,2-Bis(4-fluorophenyl)-6-({*N*-[(9*H*-fluoren-9-yl)methoxy]carbamoyl}methyl)-

2*H*-1-benzopyran-3-carboxylic acid (**6**) S 33 – S 34

Spectroscopic data

UV-vis spectra S 35 – S 40

^1^H NMR and ^13^C NMR spectra S 41 – S 63

ORTEP plots of compounds **1**, **3** and **5a**[[2]](#footnote-3) S 64 – S 66

References S 67

# Part II Time-resolved photophysical spectroscopy

Materials and Methods S 68 – S 69

References S 69

**Part I Synthesis and characterization**

**General notes:** All starting materials and reagents were purchased in the highest available purity and were used without further purification. All reactions were conducted in flame-dried glassware under an atmosphere of dry nitrogen. Solvents were dried prior to use according to standard procedures [1]. Kugelrohr distillations were performed on a Büchi KGR-51 Kugelrohr oven. Thin layer chromatography (silica, Merck 60 F_254_ plates) was visualized by UV light or potassium permanganate stain. Preparative flash-chromatography was performed on ICN Biomedicals GmbH silica gel 60 (32 – 63 *μ*m, 60 Å). Melting points were recorded on a melting point apparatus using open capillary tubes and remain uncorrected. Infrared spectra were recorded as ATR (attenuated total reflectance) on a Nicolet FT-IR 750 spectrometer and are reported as wavenumbers in cm^−1^. NMR spectra were recorded on Bruker AC 200, AM 400 or DRX 500 spectrometers. The frequencies used for ^1^H NMR and ^13^C NMR spectra are mentioned for the particular substances. Chemical shifts are given as dimensionless *δ* values and coupling constants in Hz. Solvents are mentioned for the particular substances; their residual signals were used as internal standard. The number of protons was determined by integrating the corresponding signals. The samples for mass spectra and high-resolution MS (Finnigan MAT 95 SQ or Varian MAT 711 spectrometer) were ionized by EI at 70 eV, the temperatures used to evaporate the samples are given for the particular substance. Elemental analysis (Anal.) were recorded on an Analytik Jena Elementar Vario EL. UV-vis spectra were recorded on a Shimadzu UV-Visible Spectrophotometer UV-1601 at a concentration of
2.5 × 10^−5^ m in HPLC grade acetonitrile or spectroscopic grade dichloromethane at rt. Analytical reversed phase (RP) high-performance liquid chromatography was performed on the following experimental setup: a Bio-Tek System 525 as pump, an Autosampler 525 Kontron Instrument as autosampler, a Bio-Tek Diodenarraydetektor as detector and a Phenomenex Luna® as column (5 *μ*m, C18, 250 mm × 4.6 mm). The acquisition parameters are stated at the particular substance. HPLC-based ratios of conversion and purity were determined at 210 nm. The acquisition parameters are stated at the particular substance. X-ray diffraction data were collected on an Oxford Diffraction Xcalibur S diffractometer. The diffractometer was equipped with a Sapphire CCD detector and an enhanced monochromated MoKα source on a four-circle κ platform. The diffraction frames were integrated by using the CrysAlisRed program, the set of data were corrected for empirical absorption with SCALE3 ABSPACK.[[3]](#footnote-4) The structure was solved by direct methods and refined using the program SHELX97.[[4]](#footnote-5) Compound nomenclature: According to IUPAC recommendations 1998 (FR-2.2.7) [2] the compounds named on the basis of 1‑benzopyran and naphtho[2,1-*b*]pyran would have to be named as the corresponding chromene and benzo[*f*]chromene derivatives, respectively.

**Four-step Synthesis of Compound 8b – Experimental Procedures for the Preparation of the Precursors**

4-(Allyloxy)benzonitrile [3]

4-Hydroxybenzonitrile (23.8 g, 0.20 mol, 1.00 equiv) and K_2_CO_3_ (30.4 g, 0.22 mol, 1.10 equiv) were suspended in 20 mL acetone and magnetically stirred at rt for 20 min until a gummy slurry was obtained. Then allylbromide (17.3 mL, 0.22 mol, 1.10 equiv) was added in a single portion. The magnetically stirred slurry was gently heated to reflux for 16 h under an ambient atmosphere until TLC monitoring indicated complete consumption of the 4-hydroxybenzonitrile. The slurry was cooled to rt and diluted with 500 mL of diethyl ether and 200 mL of H_2_O. After separation of the organic layer the water phase was extracted with diethyl ether (5 × 200 mL). The combined organic phases were successively washed with water (150 mL) and brine (2 × 150 mL) and were subsequently dried (MgSO_4_). Evaporation of the solvent afforded 4-(allyloxy)benzonitrile in 92% yield (29.3 g, 0.19 mol) as a colorless solid. mp: 42 – 43 °C (lit. 43 – 44 °C [4]). *R_f_ =* 0.63 (pentane/diethyl ether, 1/2). ^1^H NMR (200 MHz, CDCl_3_): *δ* = 7.64 – 7.50 (m, 2 H), 6.99 – 6.89 (m, 2 H), 6.12 – 5.93 (m,
1 H), 5.47 – 5.29 (m, 2 H), 4.60 – 4.56 (m, 2 H). ^13^C NMR (50 MHz, CDCl_3_):*  δ* = 162.0 (Cq), 134.1 (CH), 132.2 (CH), 119.4 (Cq), 118.7 (CH_2_), 115.6 (CH), 104.3 (Cq), 69.2 (CH_2_). IR: (*ν*) = 3083 (w), 3025 (w), 2985 (w), 2925 (w), 2873 (w), 2224 (s), 1605 (s), 1508 (s), 1257 (s), 1173 (s), 995 (m), 834 (m). MS (rt): *m/z* (%) = 160 (10) [M+H]^+^, 159 (100) [M]^+^, 119 (25), 102 (10), 90 (12). HRMS calcd for C_10_H_9_NO, *m/z*: 159.0684; found: 159.0681. Anal. calcd for C_10_H_9_NO C 75.45%, H 5.70%, N 8.80%; found: C 75.43%, H 5.70%, N 8.79%.

4-(Allyloxy)benzylamine [5]

LiAlH_4_ (14.4 g, 0.38 mol, 4.00 equiv) was suspended at 0 – 5 °C in 350 mL of diethyl ether. Then 4‑(allyloxy)benzonitrile (29.3 g, 0.19 mol, 1.00 equiv), dissolved in 150 mL diethyl ether, was added dropwise within 90 min by means of a double tip needle. The suspension was gently warmed to rt and after 30 min TLC monitoring indicated the complete consumption of the nitrile. Unreacted LiAlH_4_ was carefully neutralized by adding 60 mL of 2-propanol. The reaction mixture was poured into 2.5 L of water and the layers were separated. The aqueous layer was subsequently extracted with dichloromethane (6 × 500 mL). The combined organic layers were dried (MgSO_4_) and concentrated in vacuo, affording the pure amine in 83% yield (25.7 g, 0.16 mol) as a colorless liquid. *R_f_ =* 0.36 (acetone + 1 vol% NEt_3_). ^1^H NMR (200 MHz, CDCl_3_):* δ* = 7.21 – 7.14 (m, 2 H), 6.88 – 6.81 (m, 2 H), 6.11 – 5.92 (m, 1 H), 5.43 – 5.21 (m, 2 H), 4.50 – 4.45 (m, 2 H), 3.75 (s,
2 H), 1.34 (s, 2 H). ^13^C NMR (50 MHz, CDCl_3_): *δ* = 157.6 (Cq), 135.7 (Cq), 133.4 (CH), 128.3 (CH), 117.6 (CH_2_), 114.8 (CH), 68.9 (CH_2_), 45.9 (CH_2_). IR: (*ν*) = 3332 (br, w), 3079 (w), 3063 (w), 3030 (w), 2991 (w), 2911 (m), 2862 (m), 2625 (w), 1662 (s), 1611 (s), 1510 (s), 1369 (s), 1241 (s), 1109 (w), 1024 (m), 925 (m), 825 (m). MS (rt): *m/z* (%) = 164 (10) [M+H]^+^, 163 (100) [M]^+^, 162 (95), 145 (28), 122 (92), 106 (38), 94 (44). HRMS calcd for C_10_H_13_NO, *m/z*: 163.0997; found: 163.0991. Anal. calcd for C_10_H_13_NO C 73.59%, H 8.03%, N 8.58%; found: C 73.42%,
H 7.88%, N 8.40%.

(9*H*-Fluoren-9-yl)methyl [4-(allyloxy)benzyl]carbamate [6]

4‑(Allyloxy)benzylamine (20.0 g, 0.12 mol, 1.00 equiv) was dissolved in 300 mL of THF/water (1/1). Then NaHCO_3_ (13.4 g, 0.16 mol, 1.30 equiv) was added in a single portion. The resulting solution was stirred at rt for 10 min and *N*-[(9*H*-fluoren-9‑yl)­methoxy­carbonyloxy]succinimide (41.3 g, 0.12 mol, 1.00 equiv) was added portionwise within 1 h. The reaction mixture was stirred at rt until TLC monitoring indicated complete consumption of the starting material (13 h). The reaction mixture was diluted with 600 mL of water and extracted with diethyl ether (3 × 200 mL). The combined organic phases were washed with water (2 × 75 mL), then dried (MgSO_4_) and concentrated in vacuo. The carbamate was obtained in quantitative yield (46.2 g, 0.12 mol) as colorless solid. mp: 108 – 109 °C. *R_f_ =* 0.32 (pentane/diethyl ether, 1/1). ^1^H NMR (200 MHz, CDCl_3_):[[5]](#footnote-6)
*δ* = 7.79 – 7.75 (m, 2 H), 7.62 – 7.58 (m, 2 H), 7.45 – 7.31 (m, 4 H), 7.21 – 7.17 (m,
2 H), 6.90 – 6.86 (m, 2 H), 6.16 – 5.97 (m, 1 H), 5.47 – 5.26 (m, 2 H), 5.03 (br s, 1 H),
4.56 – 4.51 (m, 2 H), 4.46 (d, *J* = 6.8 Hz, 2 H), 4.33 – 4.30 (m, 2 H), 4.23 (t, *J* = 6.9 Hz, 1 H). ^13^C NMR (50 MHz, CDCl_3_):[[6]](#footnote-7) *δ* = 158.2 (Cq), 156.5 (Cq), 144.1 (Cq), 141.5 (Cq), 133.3 (CH), 130.8 (Cq), 129.0 (CH), 127.8 (CH), 127.2 (CH), 125.2 (CH), 120.1 (CH), 117.9 (CH_2_), 115.0 (CH), 69.0 (CH_2_), 66.7 (CH_2_), 47.4 (CH), 44.7 (CH_2_). IR: (*ν*) = 3329 (br, m), 3064 (w), 3039 (w), 3017 (w), 2939 (w), 2923 (w), 2884 (w), 2862 (w), 1689 (s), 1539 (m), 1255 (m), 987 (m). MS (180 °C): *m/z* (%) = 385 (<1) [M]^+^, 207 (4), 179 (21), 178 (100), 147 (4). HRMS calcd for C_25_H_23_NO_3_, *m/z*: 385.1678; found: 385.1680. Anal. calcd for C_25_H_23_NO_3_ C 77.90%, H 6.01%,
N 3.63%; found: C 77.90%, H 5.96%, N 3.61%.

(9*H*-Fluoren-9-yl)methyl (4-hydroxybenzyl)carbamate (**8b**) [7,8]

[4-(Allyloxy)benzyl]carbamate (5.70 g, 15.0 mmol, 1.00 equiv) and Pd(PPh_3_)_4_ (347 mg, 0.3 mmol, 2 mol%) were dissolved at rt in 150 mL of degassed dichloromethane under an atmosphere of argon. Then degassed acetic acid (6.90 mL, 0.12 mol, 8.00 equiv) and degassed water (2.20 mL, 0.12 mol, 8.00 equiv) were added in a single portion. Afterwards Bu_3_Sn-H (11.9 mL, 45.0 mmol, 3.00 equiv) was added dropwise within 30 min. After additional 15 min, TLC monitoring indicated complete consumption of the starting material. The reaction mixture was concentrated to 25% of its volume in vacuo, and then pentane was added until the product started to precipitate. The precipitation was completed at –25 °C for 12 h. The isolated precipitate was recrystallized from toluene/cyclohexane (3/2) affording the hydroxycarbamate **8b** in quantitative yield (5.18 g, 15.0 mmol) as a colorless solid. mp:
144 – 146 °C (lit. 128 – 130 °C [6]). *R_f_ =* 0.35 (pentane/diethyl ether, 5/9). ^1^H NMR (200 MHz, CDCl_3_):[[7]](#footnote-8) *δ* = 7.78 – 7.74 (m, 2 H), 7.60 – 7.57 (m, 2 H), 7.43 – 7.30 (m, 5 H), 7.14 – 7.10 (m,
2 H), 6.80 – 6.76 (m, 2 H), 5.02 (br s, 1 H), 4.45 (d, *J* = 6.7 Hz, 2 H), 4.31 – 4.28 (m, 2 H), 4.22 (t, *J* = 6.8 Hz, 1 H). ^13^C NMR (50 MHz, acetone-*d*_6_):[[8]](#footnote-9) *δ* = 157.4 (Cq), 157.3 (Cq), 145.2 (Cq), 142.2 (Cq), 131.6 (Cq), 129.7 (CH), 128.5 (CH), 128.0 (CH), 126.2 (CH), 120.8 (CH), 116.0 (CH), 66.9 (CH_2_), 48.2 (CH), 44.8 (CH_2_). IR: (*ν*) = 3329 (br, m), 3066 (w), 3041 (w), 3020 (w), 2950 (w), 2892 (w), 1695 (s), 1516 (s), 1450 (m), 1247 (br, s), 1044 (w), 987 (w), 832 (w). MS (170 °C): *m/z* (%) = 345 (<1) [M]^+^, 180 (6), 179 (86), 178 (100), 165 (44), 107 (84). HRMS calcd for C_22_H_19_NO_3_, *m/z*: 345.1365; found: 345.1366. Anal. calcd for C_22_H_19_NO_3_ C 76.50%, H 5.54%, N 4.06%; found: C 76.47%, H 5.53%, N 3.96%.

1,1-Bis(4-fluorophenyl)prop-2-yn-1-ol (**9**)

4,4’-Difluorobenzophenone (21.8 g, 0.10 mol, 1.00 equiv) was dissolved at rt in 200 mL of THF. Then 400 mL of a 0.5 m ethynylmagnesium chloride solution (0.20 mmol, 2.00 equiv) were added dropwise within 12 h. After complete addition, the reaction mixture was magnetically stirred for further 12 h at rt until TLC monitoring indicated a complete consumption of the starting material. The solution was diluted with 750 mL of diethyl ether and carefully hydrolysed with 200 mL of a saturated NH_4_Cl solution. The separated aqueous layer was extracted with diethyl ether (3 × 350 mL). The combined organic layers where washed with brine (2 × 150 mL), dried (MgSO_4_) and concentrated in vacuo. The crude alcohol **9** was purified by two consecutive Kugelrohr distillations (120 – 150 °C, 0.1 mbar) affording a colorless oil (17.3 g, 70.83 mmol, 71%). *R_f_ =* 0.46 (pentane/diethyl ether, 2/1). ^1^H NMR (200 MHz, CDCl_3_): *δ *= 7.59 – 7.51 (m, 4 H),
7.08 – 6.96 (m, 4 H), 2.91 (s, 1 H), 2.82 (s, 1 H). ^13^C NMR (50 MHz, CDCl_3_): *δ* = 162.5 (d,
*J*(F-C) = 247 Hz, Cq), 140.2 (d, *J*(F-C) = 3 Hz, Cq), 128.0 (d, *J*(F-C) = 8 Hz, CH), 115.3 (d,
*J*(F-C) = 22 Hz, CH), 86.0 (Cq), 76.1 (CH), 73.5 (Cq). IR: (*ν*) = 3563 (br, m), 3412 (br, m), 3300 (s), 3117 (w), 3075 (w), 3048 (w), 2978 (w), 2933 (w), 2885 (w), 2119 (w), 2034 (w), 1899 (w), 1702 (m), 1603 (s), 1506 (s), 1411 (m), 1329 (m), 1224 (s), 1158 (s), 993 (s), 905 (m), 835 (s). MS (rt): *m/z* (%) = 245 (14) [M+H]^+^, 244 (100) [M]^+^, 243 (42) [M–H]^+^, 227 (23), 215 (20), 201 (28), 149 (86), 123 (39), 95 (32). HRMS calcd for C_15_H_10_F_2_O, *m/z*: 244.0700; found: 244.0691. Anal. calcd for C_15_H_10_F_2_O C 73.76%, H 4.13%; found: C 73.75%, H 4.15%.

**Experimental Procedures for the Preparation of Functionalized Benzo- and Naphthopyrans**

3,3-Bis(4-fluorophenyl)-8-(methoxycarbonyl)-3*H*-naphtho[2,1-*b*]pyran (**10**)

Methyl 6-hydroxy-2-naphthoate (**7**; 2.65 g, 13.10 mmol, 1.00 equiv) and pyridinium tosylate (166 mg, 0.66 mmol, 5 mol%) were heated to reflux in 440 mL DCE (0.03 m in naphthoate **7**). Then trimethyl orthoformate (2.84 mL, 26.20 mmol, 2.00 equiv) was added in a single portion. To this mixture the propargyl alcohol **9** (4.5 g, 18 mmol, 1.4 equiv) was added dropwise via syringe. The mixture was heated to reflux for 20 h until TLC monitoring indicated complete consumption of the starting material. The brown solution was cooled to rt and diluted with 160 mL of dichloromethane. The organic layer was washed with water (50 mL) and with brine (2 × 50 mL), concentrated in vacuo and the crude product was lyophilized. The crude product was crystallized from ethanol (about 1 mL per 20 mg) to give naphthopyran **10** as colorless micro crystals in 77% yield (4.3 g, 10.04 mmol). mp: 174 – 176 °C (decomposition). *R_f_* = 0.59 (pentane/diethyl ether, 1/1). *t*_R_ = 28.6 min (flow rate: 1 mL/min, rt; eluent: acetonitrile/water, gradient: within 25 min form 1/1 to 9/1, then pure acetonitrile). UV-vis (2.5 × 10^−5^ m, acetonitrile) *λ*_max_ (A): 334 nm (*ε* = 5104 dm^3^ × m^−1^ × cm^−1^), *λ*_max_ (B): 261 nm (*ε* = 64224 dm^3^ × m^−1^ × cm^−1^). ^1^H NMR (400 MHz, CDCl_3_): *δ* = 8.48 (s, 1 H), 8.06 (d, *J* = 8.9 Hz, 1 H), 7.98 (d, *J* = 8.8 Hz, 1 H), 7.77 (d, *J* = 8.9 Hz, 1 H), 7.44 – 7.41 (m, 4 H), 7.32 (d, *J* = 10.0 Hz, 1 H), 7.21 (d, *J* = 8.8 Hz, 1 H), 7.08 – 6.96 (m, 4 H), 6.20 (d, *J* = 10.0 Hz, 1 H), 3.96 (s, 3 H). ^13^C NMR (101 MHz, CDCl_3_):[[9]](#footnote-10) *δ* = 167.3 (Cq), 162.4 (d, *J*(F-C) = 247 Hz, Cq), 152.3 (Cq), 140.3 (d, *J*(F-C) = 3 Hz, Cq), 132.2 (Cq), 131.8 (CH), 131.7 (CH), 128.9 (d, *J*(F-C) = 8 Hz, CH), 128.6 (Cq), 127.7 (CH), 126.4 (CH), 125.6 (Cq), 121.7 (CH), 119.7 (CH), 119.2 (CH), 115.3 (d, *J*(F-C) = 21 Hz, CH), 114.0 (Cq), 82.3 (Cq), 52.3 (CH_3_). IR: (*ν*) = 3071 (w), 2975 (w), 2951 (w), 2845 (w), 1899 (w), 1716 (s), 1630 (m), 1622 (m), 1601 (m), 1506 (s), 1468 (s), 1435 (m), 1381 (m), 1281 (s), 1227 (s), 1210 (s), 1178 (s), 1158 (s), 1105 (s), 1084 (s), 1008 (s), 953 (m), 836 (s). MS (140 °C): *m/z* (%) = 428 (50) [M]^+^, 397 (4), 369 (12), 333 (90), 273 (12), 244 (12), 227 (8), 201 (14), 121 (100). HRMS calcd for C_27_H_18_F_2_O_3_, *m/z*: 428.1224; found: 428.1229. Anal. calcd for C_27_H_18_F_2_O_3_: C 75.69%, H 4.23%; found: C 75.58%, H 4.24%.

2-Bromo-3,3-bis(4-fluorophenyl)-8-(methoxycarbonyl)-2,3-dihydro-

1*H*-naphtho[2,1-*b*]pyran-1-ol (**11**)

Naphthopyran **10** (4.28 g, 10.0 mmol, 1.00 equiv) was dissolved in 60 mL DMSO (0.2 m) and magnetically stirred at rt. Water (0.4 mL, 24.0 mmol, 2.30 equiv) was added to the solution in a single portion followed by the portionwise addition of NBS (4.48 g, 19.8 mmol, 1.98 equiv) over a period of 30 min. The reaction mixture was stirred for further 60 min until only traces of starting material remained detectable by TLC monitoring. Then the reaction mixture was poured into 350 mL of water. The aqueous phase was extracted with ethyl acetate (5 × 50 mL). The combined organic layers were washed with 25 mL of water and 50 mL of brine, respectively, dried (MgSO_4_) and concentrated in vacuo. The remainder was purified by flash-chromatography (silica, hexane/diethyl ether, 5/1 to 3/1) affording bromohydrin **11** (4.5 g, 8.6 mmol, 86%) as an amorphous wax. *R_f_* = 0.26 (pentane/diethyl ether, 1/1). ^1^H NMR (400 MHz, CDCl_3_): *δ* = 8.56 (d, *J*= 1.5 Hz, 1 H), 8.10 (dd, *J* = 8.8 Hz, *J* = 1.7 Hz, 1 H), 8.06 (d, *J* = 8.9 Hz, 1 H), 8.00 (d, *J* = 9.2 Hz, 1 H), 7.74 – 7.69 (m, 2 H), 7.51 (d, *J* = 9.3 Hz, 1 H), 7.49 – 7.46 (m, 2H), 7.11 – 7.06 (m, 2 H), 6.96 – 6.91 (m, 2 H), 5.62 – 5.59 (m, 2 H), 3.97 (s, 3 H), 1.45 – 1.42(m, 1 H). ^13^C NMR (101 MHz, CDCl_3_):[[10]](#footnote-11) *δ* = 167.2 (Cq), 162.1 (d, *J*(F-C) = 249 Hz, Cq), 162.1 (d, *J*(F-C) = 249 Hz, Cq), 151.4 (Cq), 139.7 (m, Cq), 135.9 (d, *J*(F-C) = 3 Hz, Cq), 135.6 (Cq), 133.1 (CH), 131.5 (CH), 129.1 (Cq), 128.6 (d, *J*(F-C) = 9 Hz, CH), 127.1 (CH), 126.6 (d, *J*(F-C) = 6 Hz, CH), 126.2 (Cq), 123.5 (CH), 119.6 (CH), 116.0 (d, *J*(F-C) = 21 Hz, CH), 115.6 (d, *J*(F-C) = 22 Hz, CH), 113.5 (CH), 82.1 (CH), 68.4 (CH), 55.0 (CH), 52.4 (CH_3_). IR: (*ν*) = 3572 (w), 3472 (br, w), 3072 (w), 2975 (w), 2952 (w), 2846 (w), 1716 (s), 1624 (s), 1603 (m), 1507 (s), 1472 (s), 1405 (m), 1286 (s), 1237 (s), 1210 (s), 1081 (s), 999 (s), 836 (s). MS (190 °C): *m/z* (%) = 526 (<1) [M{^81^Br}]^+^, 524 (<1) [M{^79^Br}]^+^, 447 (4), 427 (8), 294 (18), 230 (36), 121 (100). HRMS calcd for C_27_H_19_BrF_2_O_4_, *m/z*: 524.0435; found: 524.0436. Anal. calcd for C_27_H_19_BrF_2_O_4_: C 61.73%, H 3.65%; found: C 61.54%, H 3.77%.

2-Bromo-3,3-bis(4-fluorophenyl)-8-(methoxycarbonyl)-3*H*-naphtho[2,1-*b*]pyran (**1**)

Bromohydrin **11** was dissolved in 30 mL of toluene (5.5 g, 10.5 mmol, 1.0 equiv; 0.4 m). 4-Toluenesulfonic acid monohydrate (303 mg, 1.6 mmol, 15 mol%) was added in a single portion and the resulting suspension was heated to reflux for 60 min until only traces of the starting material remained detectable by TLC monitoring. The reaction mixture was diluted with 60 mL of ethyl acetate, washed with water (4 × 10 mL), dried (MgSO_4_) and concentrated in vacuo. The remainder was once triturated with cold pentane and four times crystallized from ethanol/ethyl acetate (5/1; about 1 mL per 6 mg) to give naphthopyran **1** as pale pink crystals (2.9 g, 5.7 mmol, 54%). mp: 211 – 213 °C. *R_f_* = 0.32 (pentane/diethyl ether, 4/1). *t*_R_ = 19.9 min (flow rate: 1 mL/min, rt; eluent: acetonitrile/water, gradient: within 20 min from 7/3 to pure acetonitrile). UV-vis (2.5 × 10^−5^ m, acetonitrile) *λ*_max_ (A): 335 nm (*ε* = 6036 dm^3^ × m^−1^ × cm^−1^), *λ*_max_ (B): 265 nm (*ε* = 59840 dm^3^ × m^−1^ × cm^−1^). ^1^H NMR (400 MHz, CDCl_3_): *δ* = 8.47 (s, 1 H), 8.07 (d, *J* = 8.8 Hz, 1 H), 7.92 (d, *J* = 8.9 Hz, 1 H), 7.79 (s, 1 H), 7.76 (d, *J* = 8.8 Hz, 1 H), 7.49 – 7.46 (m, 4 H), 7.17 (d, *J* = 8.8 Hz, 1 H), 7.06 – 7.01 (m, 4 H), 3.96 (s, 3 H). ^13^C NMR (101 MHz, CDCl_3_):[[11]](#footnote-12) *δ* = 167.1 (Cq), 162.8 (d, *J*(F-C) = 248 Hz, Cq), 151.4 (Cq), 137.7 (d, *J*(F-C) = 4 Hz, Cq), 132.0 (CH), 131.7 (CH), 131.2 (Cq), 130.5 (d, *J*(F-C) = 8 Hz, CH), 128.8 (CH), 126.7 (CH), 126.0 (Cq), 125.0 (Cq), 121.7 (CH), 120.9 (Cq), 119.0 (CH), 115.6 (Cq), 115.0 (d, *J*(F-C) = 22 Hz, CH), 86.6 (Cq), 52.3 (CH_3_). IR: (*ν*) = 3074 (w), 2975 (w), 2951 (w), 2844 (w), 1718 (s), 1627 (m), 1601 (m), 1586 (w), 1506 (s), 1468 (s), 1436 (w), 1384 (w), 1272 (s), 1230 (s), 1201 (s), 1160 (m), 1086 (m), 1010 (s), 961 (w), 875 (w), 837 (s). MS (170 °C): *m/z* (%) = 508 (<1) [M{^81^Br}]^+^, 506 (<1) [M{^79^Br}]^+^, 427 (100), 367 (3), 338 (3), 198 (12). HRMS calcd for C_27_H_17_BrF_2_O_3_, *m/z*: 506.0329; found: 506.0325. Anal. calcd for C_27_H_17_BrF_2_O_3_: C 63.92%, H 3.38%; found: C 63.94%, H 3.46%.

2,2-Bis(4-fluorophenyl)-6-[(methoxycarbonyl)methyl]-2*H*-1-benzopyran (**12a**)

Methyl 2-(4-hydroxyphenyl)acetate (**8a**; 1.5 g, 9.00 mmol, 1.00 equiv) and pyridinium tosylate (113 mg, 0.45 mmol, 5 mol%) were heated to reflux in 30 mL of DCE (0.5 m in acetate **8a**). Then trimethyl orthoformate was added in a single portion (1.97 mL, 19.00 mmol, 2.12 equiv). To this mixture the propargyl alcohol **9** (2.2 g, 9.00 mmol, 1.00 equiv) was added dropwise via a syringe. The brown solution was heated to reflux for 5 h, cooled to rt and diluted with 100 mL of dichloromethane. The organic layer was washed with water (2 × 25 mL) and with brine (20 mL), dried (MgSO_4_) and concentrated in vacuo. Purification by flash-chromatography (silica, pentane/diethyl ether, 3/1) gave benzopyran **12a** in 23% yield
(812 mg, 2.10 mmol) as a colorless solid. mp: 26 – 28 °C. *R_f_* = 0.67 (pentane/ethyl acetate, 1/1). *t*_R_ = 12.1 min (flow rate: 1 mL/min, rt; eluent: acetonitrile/water, gradient: within 20 min from 7/3 to pure acetonitrile). UV-vis (2.5 × 10^−5^ m, acetonitrile) *λ*_max_ (A): 316 nm (*ε* = 3480 dm^3^ × m^−1^ × cm^−1^), *λ*_max_ (B): 273 nm (*ε* = 8076 dm^3^ × m^−1^ × cm^−1^). ^1^H NMR (400 MHz, CDCl_3_): *δ* = 7.36 – 7.34 (m, 4 H), 7.05 – 6.94 (m, 6 H), 6.85 – 6.83 (m, 1 H), 6.60 (d, *J* = 9.8 Hz, 1 H), 6.07 (d, *J* = 9.8 Hz, 1 H), 3.68 (s, 3 H), 3.50 (s, 2 H). ^13^C NMR (101 MHz, CDCl_3_):[[12]](#footnote-13)
*δ* = 172.3 (Cq), 162.3 (d, *J*(F-C) = 247 Hz, Cq), 151.4 (Cq), 140.6 (d, *J*(F-C) = 3 Hz, Cq), 130.7 (CH), 128.9 (d, *J*(F-C) = 8 Hz, CH), 128.8 (CH), 127.6 (CH), 127.0 (Cq), 123.7 (CH), 121.1 (Cq), 116.7 (CH), 115.2 (d, *J*(F-C) = 21 Hz, CH), 82.0 (Cq), 52.2 (CH_3_), 40.4 (CH_2_). IR: (*ν*) = 3068 (w), 3043 (w), 3000 (w), 2952 (w), 2844 (w), 1736 (s), 1636 (w), 1601 (m), 1506 (s), 1435 (m), 1227 (s), 1158 (s), 991 (m), 835 (s). MS (120 °C): *m/z* (%) = 392 (70) [M]^+^, 333 (14), 297 (100), 244 (52). HRMS calcd for C_24_H_18_F_2_O_3_, *m/z*: 392.1224; found: 392.1223. Anal. calcd for C_24_H_18_F_2_O_3_ C 73.46%, H 4.62%; found: C 73.22%, H 4.62%.

2,2-Bis(4-fluorophenyl)-6-({[(9*H*-fluoren-9-yl)methoxycarbonyl]amino}methyl)-2*H*‑1‑benzopyran (**12b**) [9]

(Hydroxybenzyl)carbamate **8b** (4.80 g, 14.00 mmol, 1.00 equiv) and pyridinium tosylate (176 mg, 0.70 mmol, 5 mol%) were heated to reflux in 70 mL DCE (0.2 m in carbamate **8b**). Then trimethyl orthoformate was added in a single portion (3.10 mL, 28.00 mmol, 2.00 equiv). To this mixture the propargyl alcohol **9** (3.80 g, 15.40 mmol, 1.10 equiv) was added dropwise via syringe. The brown solution was heated to reflux for 5 h, cooled to rt and diluted with 200 mL of dichloromethane. The organic layer was washed with water (2 × 25 mL) and with brine (50 mL), dried (MgSO_4_) and concentrated in vacuo. The crude product was purified by flash-chromatography (silica, pentane/diethyl ether, 10/1). Benzopyran **12b** was isolated in 25% yield (2.0 g, 3.5 mmol) as a colorless solid. mp: 95 – 97 °C. *R_f_ =* 0.37 (pentane/dichloromethane, 2/1). UV-vis (1.0 × 10^−4^ m; dichloromethane, spectroscopic grade): *λ*_max_ (A): 312 nm (*ε* = 1869 dm^3^ × m^−1^ × cm^−1^), *λ*_max_ (B): 301 nm (*ε* = 5594 dm^3^ × m^−1^ × cm^−1^), *λ*_max_ (C): 292 nm (*ε* = 4538 dm^3^ × m^−1^ × cm^−1^). ^1^H NMR (500 MHz, CDCl_3_):[[13]](#footnote-14) *δ* = 7.78 – 7.76 (m, 2 H), 7.59 – 7.58 (m, 2 H), 7.42 – 7.36 (m, 5 H), 7.32 – 7.29 (m, 2 H), 7.03 – 7.00 (m, 5 H), 6.95 (s, 1 H), 6.86 – 6.85 (m, 1 H), 6.60 (d, *J* = 9.8 Hz, 2 H), 6.10 (d, *J* = 9.8 Hz, 1 H), 5.01 (br s, 1 H), 4.45 (d, *J* = 6.8 Hz, 2 H), 4.26 – 4.25 (m, 2 H), 4.22 (t, *J* = 7.0 Hz, 1 H). ^13^C NMR (126 MHz, CDCl_3_):[[14]](#footnote-15) *δ* = 162.3 (d, *J*(F-C) = 248 Hz, Cq), 156.5 (Cq), 151.7 (Cq), 144.0 (Cq), 141.5 (Cq), 140.5 (d, *J*(F-C) = 3 Hz, Cq), 131.6 (Cq), 129.2 (CH), 129.0 (CH), 128.9 (d, *J*(F-C) = 8 Hz, CH), 127.8 (CH), 127.2 (CH), 126.2 (CH), 125.1 (CH), 123.7 (CH), 121.2 (Cq), 120.1 (CH), 116.8 (CH), 115.2 (d, *J*(F-C) = 22 Hz, CH), 82.0 (Cq), 66.8 (CH_2_), 47.4 (CH), 44.7 (CH_2_). IR: (*ν*) = 3328 (br, w), 3066 (w), 3042 (w), 2971 (w), 2943 (w), 2889 (w), 1703 (s), 1601 (m), 1507 (s), 1407 (w), 1325 (w), 1227 (s), 1127 (m), 835 (m). MS (240 °C): *m/z* (%) = 571 (8) [M]^+^, 476 (12), 393 (32), 336 (30), 178 (100), 159 (20). HRMS calcd for C_37_H_27_F_2_NO_3_, *m/z*: 571.1959; found: 571.1955. Anal. calcd for C_37_H_27_F_2_NO_3_ C 77.74%, H 4.76%, N 2.45%; found: C 77.43%, H 4.73%, N 2.40%.

3-Bromo-2,2-bis(4-fluorophenyl)-6-[(methoxycarbonyl)methyl]-3,4-dihydro-2*H*‑1‑benzopyran‑4‑ol (**13a**)

Benzopyran **12a** (804 mg, 2.05 mmol, 1.00 equiv) was dissolved in 35 mL of DMSO (0.06 m) and magnetically stirred at rt. Water (74 *µ*l, 4.10 mmol, 2.00 equiv) was added to the solution in a single portion followed by the portionwise addition of NBS (927 mg, 4.10 mmol, 2.00 equiv) over a period of 30 min. The reaction mixture was stirred for further 60 min until only traces of the starting material remained detectable by TLC monitoring. Then, the reaction mixture was poured into 150 mL of water. The aqueous phase was extracted with ethyl acetate (3 × 75 mL). The combined organic layers were washed with 25 mL water of and 25 mL of brine, respectively, dried (MgSO_4_) and concentrated in vacuo. The crude product was purified by flash-chromatography (silica, pentane/ethyl acetate, 5/2) affording bromohydrin **13a** as a colorless solid (763 mg, 1.56 mmol, 76%). mp: 65 – 68 °C. *R_f_* = 0.53 (pentane/ethyl acetate, 1/1). ^1^H NMR (400 MHz, CDCl_3_): *δ* = 7.74 – 7.70 (m, 2 H), 7.39 – 7.34 (m, 2 H), 7.28 – 7.27 (m, 1 H), 7.25 – 7.23 (m, 1 H), 7.09 – 6.94 (m, 5 H), 5.18 – 5.17 (m, 1 H), 5.10 – 5.06 (m, 1 H), 3.69 (s, 3 H), 3.55 (s, 2 H), 2.02 – 2.00 (m, 1 H). ^13^C NMR (101 MHz, CDCl_3_):[[15]](#footnote-16) *δ* = 172.2 (Cq), 162.4 (d, *J*(F-C) = 248 Hz, Cq), 162.2 (d, *J*(F-C) = 248 Hz, Cq), 151.1 (Cq), 137.7 (d, *J*(F-C) = 3 Hz, Cq), 137.4 (d, *J*(F-C) = 3 Hz, Cq), 131.5 (CH), 130.3 (CH), 129.1 (d, *J*(F-C) = 8 Hz, CH), 128.6 (d, *J*(F-C) = 8 Hz, CH), 127.9 (Cq), 122.4 (Cq), 117.8 (CH), 115.6 (d, *J*(F-C) = 21 Hz, CH), 115.5 (d, *J*(F-C) = 21 Hz, CH), 83.1 (Cq), 70.2 (CH), 58.8 (CH), 52.2 (CH_3_), 40.3 (CH_2_). IR: (*ν*) = 3409 (br, w), 3069 (w), 2952 (w), 2929 (w), 2854 (w), 1735 (m), 1602 (m), 1507 (s), 1498 (s), 1437 (m), 1231 (s), 1162 (s), 1015 (m), 839 (m). MS (180 °C): *m/z* (%) = 490 (4) [M{^81^Br}]^+^, 488 (4) [M{^79^Br}]^+^, 473 (16), 471 (16), 391 (80), 331 (10), 296 (100), 215 (42), 194 (26). HRMS calcd for C_24_H_19_BrF_2_O_4_, *m/z*: 488.0435; found: 488.0434. Anal. calcd for C_24_H_19_BrF_2_O_4_: C 58.91%, H 3.91%; found: C 58.56%, H 3.98%.

3-Bromo-6-({[(9*H*-fluoren-9-yl)methoxycarbonyl]amino}methyl)-2,2-bis(4-fluorophenyl)-3,4‑dihydro-2*H*-1-benzopyran-4-ol (**13b**) [10]

Benzopyran **12b** (2.00 g, 3.50 mmol, 1.00 equiv) was dissolved in 35 mL of DMSO (0.1 m) and magnetically stirred at rt. Water (126 *µ*L, 7.00 mmol, 2.00 equiv) was added to the solution in a single portion followed by the portionwise addition of NBS (1.3 g, 7.00 mmol, 2.00 equiv) over a period of 30 min. The reaction mixture was stirred for further 60 min until only traces of starting material remained detectable by TLC monitoring. Then the reaction mixture was poured into 150 mL of water. The aqueous phase was extracted with ethyl acetate (3 × 75 mL). The combined organic layers were washed with 25 mL of water and 25 mL of brine, respectively, dried (MgSO_4_) and concentrated in vacuo. Purification was effected by flash-chromatography (silica, pentane/ethyl acetate, 2/1) affording bromohydrin **13b** in 90% yield (2.10 g, 3.14 mmol) as a colorless solid. mp: 107 – 109 °C. *R_f_ =* 0.72 (ethyl acetate). ^1^H NMR (200 MHz, CDCl_3_): *δ* = 7.78 – 7.69 (m, 4 H), 7.60 – 7.56 (m, 2 H), 7.44 – 7.19 (m, 7 H), 7.10 – 6.93 (m, 5 H), 5.19 – 5.16 (m, 1 H), 5.10 – 5.02 (m, 2 H), 4.45 (d, *J* = 6.8 Hz, 2 H), 4.31 – 4.28 (m, 2 H), 4.22 (t, *J* = 6.7 Hz, 1 H), 2.06 – 2.02 (m, 1 H). ^13^C NMR (50 MHz, CDCl_3_):[[16]](#footnote-17) *δ* = 162.3 (d, *J*(F-C) = 247 Hz, Cq), 162.2 (d, *J*(F-C) = 248 Hz, Cq), 156.5 (Cq), 151.4 (Cq), 144.0 (Cq), 141.4 (Cq), 137.6 (d, *J*(F-C) = 3 Hz, Cq), 137.4 (d, *J*(F‑C) = 3 Hz, Cq), 132.3 (Cq), 129.9 (CH), 129.1 (d, *J*(F-C) = 8 Hz, CH), 128.6 (d, *J*(F-C) = 8 Hz, CH), 127.8 (CH), 127.2 (CH), 125.1 (CH), 122.6 (Cq), 122.5 (CH), 120.1 (CH), 117.9 (CH), 115.5 (d, *J*(F-C) = 22 Hz, CH), 115.4 (d, *J*(F-C) = 22 Hz, CH), 83.2 (Cq), 70.1 (CH), 66.8 (CH_2_), 58.8 (CH), 47.3 (CH), 44.5 (CH_2_). IR: (*ν*) = 3413 (br, w), 3345 (br, w), 3066 (w), 3051 (w), 3018 (w), 2972 (w), 2948 (w), 2891 (w), 1702 (s), 1602 (m), 1508 (s), 1451 (m), 1410 (w), 1361 (w), 1228 (s), 1122 (m), 1015 (m), 839 (m). MS (210 °C): *m/z* (%) = 651 (<1) [M{^81^Br}–H_2_O]^+^, 649 (<1) [M{^79^Br}–H_2_O]^+^, 570 (17), 476 (5), 393 (15), 392 (21), 348 (12), 178 (100), 165 (18). HRMS calcd for C_37_H_26_BrF_2_NO_3_ [M–H_2_O]^+^, *m/z*: 649.1064; found: 649.1066. Anal. calcd for C_37_H_28_BrF_2_NO_4_: C 66.47%, H 4.22%, N 2.10%; found: C 66.07%, H 4.40%, N 2.23%.

3-Bromo-2,2-bis(4-fluorophenyl)-6-[(methoxycarbonyl)methyl]-2*H*-1-benzopyran (**2a**)

Bromohydrin **13a** was dissolved in 15 mL of toluene (489 mg, 1.00 mmol, 1.00 equiv; 0.07 m). 4-Toluenesulfonic acid (28.5 mg, 0.15 mmol) was added in a single portion and the resulting suspension was heated to reflux for 1.5 h until only traces of the starting material remained detectable by TLC monitoring. The reaction mixture was diluted with 40 mL of ethyl acetate, washed with water (3 × 10 mL), dried (MgSO_4_) and concentrated in vacuo. The remainder was purified by flash-chromatography (silica, pentane/diethyl ether, 3/1) affording benzopyran **2a** as a colorless solid (424 mg, 0.90 mmol, 90%). mp: 107 – 109 °C. *R_f_* = 0.58 (pentane/ethyl acetate, 2/1). *t*_R_ = 14.7 min (flow rate: 1 mL/min, rt; eluent: acetonitrile/water, gradient: within 20 min from 7/3 to pure acetonitrile). UV-vis (2.5 × 10^−5^ m, acetonitrile) *λ*_max_ (A): 312 nm (*ε* = 2580 dm^3^ × m^−1^ × cm^−1^), *λ*_max_ (B): 265 nm (*ε* = 6220 dm^3^ × m^−1^ × cm^−1^). ^1^H NMR (400 MHz, CDCl_3_): *δ* = 7.45 – 7.39 (m, 4 H), 7.12 (s, 1 H), 7.06 – 6.99 (m, 5 H), 6.93 – 6.92 (m, 1 H), 6.82 – 6.80 (m, 1 H), 3.68 (s, 3 H), 3.50 (s, 2 H). ^13^C NMR (101 MHz, CDCl_3_):[[17]](#footnote-18) *δ* = 172.1 (Cq), 162.7 (d, *J*(F-C) = 248 Hz, Cq), 150.5 (Cq), 137.8 (d, *J*(F-C) = 3 Hz, Cq), 130.9 (CH), 130.6 (d, *J*(F-C) = 8 Hz, CH), 128.9 (CH), 127.8 (Cq), 126.7 (CH), 122.5 (Cq), 122.1 (Cq), 117.1 (CH), 114.9 (d, *J*(F-C) = 22 Hz, CH), 86.3 (Cq), 52.2 (CH_3_), 40.3 (CH_2_). IR: (*ν*) = 3068 (w), 3050 (w), 3000 (w), 2951 (w), 2843 (w), 1737 (s), 1601 (m), 1507 (s), 1490 (s), 1436 (w), 1408 (w), 1340 (w), 1230 (s), 1159 (s), 1015 (m), 991 (m), 954 (w), 836 (s). MS (160 °C): *m/z* (%) = 472 (4) [M{^81^Br}]^+^, 470 (4) [M{^79^Br}]^+^, 392 (37), 391 (100), 332 (12). HRMS calcd for C_24_H_17_BrF_2_O_3_, *m/z*: 470.0329; found: 470.0322. Anal. calcd for C_24_H_17_BrF_2_O_3_: C 61.16%, H 3.64%; found: C 60.98%, H 3.65%.

3-Bromo-2,2-bis(4-fluorophenyl)-6-({[(9*H*-fluoren-9-yl)methoxycarbonyl]amino}methyl)-2*H*-1-benzopyran (**2b**) [10]

Bromohydrin **13b** was dissolved in 15 mL of toluene (1.7 g, 2.50 mmol, 1.00 equiv; 0.2 m). 4-Toluenesulfonic acid monohydrate (48 mg, 0.25 mmol, 10 mol%) was added in a single portion and the resulting suspension was heated to reflux for 60 min until only traces of the starting material remained detectable by TLC monitoring. The reaction mixture was diluted with 40 mL of ethyl acetate, washed with water (3 × 10 mL), dried (MgSO_4_) and concentrated in vacuo. The crude product was purified by flash-chromatography (silica, pentane/diethyl ether, 1/1) affording compound **2b** in 92% yield (1.5 g, 2.30 mmol) as a colorless solid. mp: 96 – 98 °C. *R_f_ =* 0.37 (pentane/ethyl acetate, 2/1). *t*_R_= 19.9 min (flow rate: 1 mL/min, rt; eluent: acetonitrile/water, gradient: within 20 min from 7/3 to pure acetonitrile). UV-vis (2.5 × 10^−5^ m, acetonitrile) *λ*_max_ (A): 316 nm (*ε* = 3364 dm^3^ × m^−1^ × cm^−1^), *λ*_max_ (B): 300 nm (*ε* = 7608 dm^3^ × m^−1^ × cm^−1^),* λ*_max_ (C): 265 nm (*ε *= 24204 dm^3^ × m^−1^ × cm^−1^). ^1^H NMR (200 MHz, CDCl_3_):[[18]](#footnote-19) *δ* = 7.78 – 7.74 (m, 2 H), 7.59 – 7.55 (m, 2 H), 7.45 – 7.29 (m, 8 H), 7.11 – 6.97 (m, 6 H), 6.90 – 6.78 (m, 2 H), 4.97 (br s, 1 H), 4.47 – 4.44 (m, 2 H), 4.25 – 4.17 (m, 3 H). ^13^C NMR (50 MHz, CDCl_3_):[[19]](#footnote-20) *δ* = 162.7 (d, *J*(F-C) = 248 Hz, Cq), 156.5 (Cq), 150.7 (Cq), 144.0 (Cq), 141.5 (Cq), 137.8 (d, *J*(F-C) = 3 Hz, Cq), 132.4 (Cq), 130.6 (d, *J*(F-C) = 8 Hz, CH), 129.4 (CH), 128.9 (CH), 127.8 (CH), 127.2 (CH), 125.2 (CH), 125.1 (CH), 122.6 (Cq), 122.3 (Cq), 120.1 (CH), 117.2 (CH), 115.0 (d, *J*(F-C) = 21 Hz, CH), 86.3 (Cq), 66.8 (CH_2_), 47.4 (CH), 44.5 (CH_2_). IR: (*ν*) = 3419 (br, w), 3330 (br, w), 3066 (w), 3043 (w), 2971 (w), 2944 (w), 2892 (w), 1703 (s), 1601 (m), 1507 (s), 1489 (s), 1450 (m), 1361 (m), 1300 (m), 1229 (s), 1159 (s), 1130 (m), 1043 (m), 990 (s), 899 (w), 836 (s). MS (210 °C): *m/z* (%) = 649 (<1) [M]^+^, 570 (20), 503 (5), 429 (12), 392 (20), 374 (28), 348 (36), 281 (18), 221 (34), 178 (100), 165 (36), 147 (32), 120 (12). HRMS calcd for C_37_H_26_BrF_2_NO_3_, *m/z*: 649.1064; found: 649.1070. Anal. calcd for C_37_H_26_BrF_2_NO_3_: C 68.32%, H 4.03%, N 2.15%; found: C 67.92%, H 4.24%, N 2.20%.

2-Cyano-3,3-bis(4-fluorophenyl)-8-(methoxycarbonyl)-3*H*-naphtho[2,1-*b*]pyran (**3**)

A Schlenk flask was charged with zinc powder (14 mg, 0.21 mmol, 0.4 equiv), iodine (23 mg, 9.1 × 10^−2^ mmol, 0.2 equiv) and 1 mL of NMP. The suspension was magnetically stirred at rt until decoloration. **CAUTION!**[[20]](#footnote-21) After a iodine-starch test indicated the absence of iodine, zinc cyanide (65 mg, 0.55 mmol, 1.12 equiv) was added followed by naphthopyran **1** (248 mg, 0.49 mmol, 1.00 equiv), [*t*‑Bu_3_PH]BF_4_ (12 mg, 0.04 mmol, 8 mol%) and Pd(dba)_2_ (12 mg, 0.02 mmol, 4 mol%). The suspension was degassed and stirred for 41 h at 45 °C until RP-HPLC monitoring indicated a complete consumption of the starting material. Then, 10 mL of ethyl acetate and 5 mL of brine were added. The layers were separated and the aqueous layer was extracted with ethyl acetate (5 × 5 mL). The combined organic phases were once washed with water (5 mL), dried (MgSO_4_) and concentrated in vacuo. The oily remainder was purified by flash-chromatography (silica, pentane/diethyl ether, 3/1) affording the nitrile **3** as a yellow solid (185 mg, 0.41 mmol, 83% yield). mp: 209 – 210 °C. *R_f_* = 0.73 (pentane/diethyl ether, 1/2). *t*_R_ = 14.4 min (flow rate: 1 mL/min, rt; eluent: acetonitrile/water, gradient: isocratic for 20 min at 17/3 then within 10 min to pure acetonitrile). UV-vis (2.5 × 10^−5^ m, acetonitrile) *λ*_max_ (A): 370 nm (*ε* = 7480 dm^3^ × m^−1^ × cm^−1^), *λ*_max_ (B): 341 nm (*ε* = 6724 dm^3^ × m^−1^ × cm^−1^), (C): 326 nm (*ε* = 6872 dm^3^ × m^−1^ × cm^−1^), *λ*_max_ (D): 262 nm (*ε* = 38820 dm^3^ × m^−1^ × cm^−1^). ^1^H NMR (400 MHz, CDCl_3_): *δ* = 8.50 (d, *J* = 1.7 Hz, 1 H), 8.15 (dd, *J* = 8.8 Hz, *J* = 1.8 Hz, 1 H), 8.08 (s, 1 H), 7.96 (d, *J* = 9.1 Hz, 1 H), 7.92 (d, *J* = 8.9 Hz, 1 H), 7.47 – 7.43 (m, 4 H), 7.23 (d, *J* = 8.9 Hz, 1 H), 7.09 – 7.03 (m, 4 H), 3.97 (s, 3 H). ^13^C NMR (101 MHz, CDCl_3_):[[21]](#footnote-22) *δ* = 166.8 (Cq), 163.1 (d, *J*(F-C) = 249 Hz, Cq), 153.9 (Cq), 136.9 (d, *J*(F‑C) = 3 Hz, Cq), 135.6 (CH), 134.7 (CH), 132.1 (Cq), 131.9 (CH), 129.8 (d, *J*(F-C) = 9 Hz, CH), 128.8 (Cq), 128.0 (CH), 126.9 (Cq), 121.5 (CH), 119.0 (CH), 117.5 (Cq), 115.6 (d, *J*(F-C) = 22 Hz, CH), 113.2 (Cq), 109.6 (Cq), 83.0 (Cq), 52.5 (CH_3_). IR: (*ν*) = 3072 (w), 2953 (w), 2926 (w), 2852 (w), 2210 (m), 1720 (s), 1626 (s), 1601 (m), 1508 (s), 1466 (m), 1281 (s), 1229 (s), 1204 (s), 1160 (s), 1088 (m), 1013 (m), 838 (s). MS (200 °C): *m/z* (%) = 455 (11) [M+2H]^+^, 454 (52) [M+H]^+^, 453 (97) [M]^+^, 421 (78), 392 (12), 358 (100), 333 (10), 201 (16). HRMS calcd for C_28_H_17_F_2_NO_3_, *m/z*: 453.1177; found: 453.1178. Anal. calcd for C_28_H_17_F_2_NO_3_: C 74.17%, H 3.78%, N 3.09%; found: C 74.06%, H 4.00%, N 3.01%.

3-Cyano-2,2-bis(4-fluorophenyl)-6-[(methoxycarbonyl)methyl]-2*H*-1-benzopyran (**5a**)

A Schlenk flask was charged with zinc powder (29 mg, 0.44 mmol, 0.4 equiv), iodine (56 mg, 0.22 mmol, 0.2 equiv) and 2.1 mL of NMP. The suspension was magnetically stirred at rt until decoloration. **CAUTION!**[[22]](#footnote-23) After a iodine-starch test indicated the absence of iodine, zinc cyanide (130 mg, 1.06 mmol, 1.05 equiv) was added followed by benzopyran **2a** (500 mg, 1.06 mmol, 1.0 equiv), [*t*‑Bu_3_PH]BF_4_ (26 mg, 0.09 mmol, 8 mol%) and Pd(dba)_2_ (25 mg, 0.04 mmol, 4 mol%). The suspension was degassed and stirred for 19 h at 45 °C until RP-HPLC monitoring indicated a complete consumption of the starting material. Then, 15 mL of ethyl acetate and 10 mL of brine were added. The layers were separated and the aqueous layer was extracted with ethyl acetate (6 × 5 mL). The combined organic phases were once washed with water (10 mL), dried (MgSO_4_) and concentrated in vacuo. The crude oil was purified by flash-chromatography (silica, pentane/diethyl ether, 1/1) to afford nitrile **5a** as a colorless solid (418 mg, 1.00 mmol, 94% yield). mp: 143 – 145 °C. *R_f_* = 0.43 (pentane/diethyl ether, 1/1). *t*_R_ = 11.2 min (flow rate: 1 mL/min, rt; eluent: acetonitrile/water, gradient: isocratic for 20 min at 17/3 then within 10 min to pure acetonitrile). UV-vis (2.5 × 10^−5^ m, acetonitrile) *λ*_max_ (A): 343 nm (*ε* = 4356 dm^3^ × m^−1^ × cm^−1^), *λ*_max_ (B): 288 nm (*ε* = 14328 dm^3^ × m^−1^ × cm^−1^). ^1^H NMR (400 MHz, CDCl_3_): *δ* = 7.42 – 7.38 (m, 5 H), 7.22 – 7.19 (m, 1 H), 7.07 – 7.03 (m, 5 H), 6.92 – 6.90 (m, 1 H), 3.69 (s, 3 H), 3.52 (s, 2 H). ^13^C NMR (101 MHz, CDCl_3_):[[23]](#footnote-24) *δ* = 171.7 (Cq), 163.0 (d, *J*(F-C) = 249 Hz, Cq), 151.9 (CH), 138.7 (Cq), 137.1 (d, *J*(F-C) = 3 Hz, Cq), 134.4 (Cq), 129.8 (d, *J*(F-C) = 8 Hz, CH), 129.0 (CH), 128.4 (CH), 119.5 (Cq), 117.7 (CH), 117.3 (Cq), 115.5 (d, *J*(F-C) = 22 Hz, CH), 111.6 (Cq), 82.7 (Cq), 52.3 (CH_3_), 40.1 (CH_2_). IR: (*ν*) = 3070 (w), 3051 (w), 3000 (w), 2953 (w), 2927 (w), 2212 (m), 1737 (s), 1623 (m), 1600 (m), 1507 (s), 1487 (m), 1436 (m), 1231 (s), 1161 (s), 997 (m), 837 (s). MS (160 °C): *m/z* (%) = 417 (60) [M]^+^, 357 (100), 322 (83). HRMS calcd for C_25_H_17_F_2_NO_3_, *m/z*: 417.1177; found: 417.1181. Anal. calcd for C_25_H_17_F_2_NO_3_: C 71.94%, H 4.11%, N 3.36%; found: C 71.78%, H 4.33%, N 3.40%.

3-Cyano-2,2-bis(4-fluorophenyl)-6-({[(9*H*-fluoren-9-yl)methoxycarbonyl]amino}methyl)-

2*H*-1-benzopyran (**5b**)

A Schlenk flask was charged with zinc powder (21 mg, 0.32 mmol, 0.4 equiv), iodine (41 mg, 0.16 mmol, 0.2 equiv) and 1.5 mL of NMP. The suspension obtained was magnetically stirred at rt until decoloration. **CAUTION!**[[24]](#footnote-25) After a iodine-starch test indicated the absence of iodine, zinc cyanide (94 mg, 0.8 mmol, 1.0 equiv) was added followed by benzopyran **2b** (0.5 g, 0.8 mmol, 1.0 equiv), [*t*‑Bu_3_PH]BF_4_ (19 mg, 0.07 mmol, 8 mol%) and 18.0 mg Pd(dba)_2_ (0.031 mmol, 3.9 mol%). The suspension was degassed and stirred for 24 h at 45 °C. After this period, RP-HPLC monitoring indicated incomplete conversion of the starting material and the reaction mixture was stirred for further 49 h at 65 °C. After this additional period, RP-HPLC monitoring indicated a constant incomplete consumption of the starting material, and 15 mL of ethyl acetate and 10 mL of brine were added. The layers were separated and the aqueous layer was extracted with ethyl acetate (6 × 5 mL). The combined organic phases were once washed with water (10 mL), dried (MgSO_4_) and concentrated in vacuo. The oily remainder was purified by flash-chromatography (silica, pentane/diethyl ether, 2/1 to 1/3) to give nitrile **5b** as a colorless, amorphous wax (320 mg, 0.54 mmol, 67% yield). *R_f_* = 0.20 (pentane/diethyl ether, 1/2). *t*_R_ = 7.7 min (flow rate: 1 mL/min, rt; eluent: acetonitrile/water, gradient: isocratic for 20 min at 17/3 then within 10 min to pure acetonitrile). UV-vis (2.5 × 10^−5^ m, acetonitrile) *λ*_max_ (A): 345 nm (*ε* = 3920 dm^3^ × m^−1^ × cm^−1^), *λ*_max_ (B): 300 nm (*ε* = 15604 dm^3^ × m^−1^ × cm^−1^), *λ*_max_ (C): 289 nm (*ε* =17464 dm^3^ × m^−1^ × cm^−1^), *λ*_max_ (D): 266 nm (*ε* = 22096 dm^3^ × m^−1^ × cm^−1^). ^1^H NMR (400 MHz, CDCl_3_):[[25]](#footnote-26),[[26]](#footnote-27) *δ* = 7.78 – 7.76 (m, 2 H), 7.58 – 7.56 (m, 2 H), 7.42 – 7.36 (m, 7 H), 7.32 – 7.28 (m, 2 H), 7.18 – 7.16 (m, 1 H), 7.09 – 7.03 (m, 5 H), 6.92 – 6.90 (m, 1 H), 5.03(br s, 1 H), 4.47 (d, *J* = 6.6 Hz, 2 H),
4.26 – 4.24 (m, 2 H), 4.20 (t, *J* = 6.6 Hz, 1 H). ^13^C NMR (101 MHz, CDCl_3_):[[27]](#footnote-28) *δ* = 163.0 (d, *J*(F-C) = 249 Hz, Cq), 156.5 (Cq), 152.2 (Cq), 143.9 (Cq), 141.5 (Cq), 138.8 (CH), 137.0 (d, *J*(F-C) = 3 Hz, Cq), 133.1 (Cq), 132.7 (CH), 129.8 (d, *J*(F-C) = 9 Hz, CH), 127.9 (CH), 127.4 (CH), 127.2 (CH), 125.1 (CH), 120.2 (Cq), 119.6 (CH), 117.8 (CH), 117.3 (Cq), 115.6 (d, *J*(F-C) = 22 Hz, CH), 111.8 (Cq), 82.7 (Cq), 66.8 (CH_2_), 47.4 (CH), 44.3 (CH_2_). IR: (*ν*) = 3485 (br, w), 3415 (br, w), 3339 (br, w), 3066 (w), 2972 (w), 2930 (w), 2893 (w), 2211 (m), 1704 (s), 1622 (m), 1606 (m), 1508 (s), 1363 (m), 1230 (s), 1160 (s), 997 (m), 838 (s). MS (270 °C): *m/z* (%) = 597 (<1) [M+H]^+^, 374 (68), 357 (77), 279 (78), 178 (100). HRMS calcd for C_38_H_27_F_2_N_2_O_3_ (M+H)^+^, *m/z*: 597.1990; found: 597.1997. Anal. calcd for C_38_H_26_F_2_N_2_O_3_: C 76.50%, H 4.39%, N 4.70%; found: C 76.17%, H 4.53%, N 4.62%.

2-[Bis(4-fluorophenyl)methyl]-7-(methoxycarbonyl)naphtho[2,1-*b*]furan (**14**) [11]

A pressure tube (Ace Glass Inc.) was charged under an atmosphere of argon with Na_2_CO_3_ (14.8 mg, 0.14 mmol, 1.00 equiv), K_4_[Fe(CN)_6_] (16.0 mg, 0.04 mmol, 0.29 equiv), Pd(OAc)_2_
(1.1 mg, 5.0 × 10^−3^ mmol, 4 mol%) and 1,1'-bis(diphenyl­phosphino)ferrocene (8.1 mg, 1.5 × 10^−2^ mmol, 10 mol%). The vessel was purged with argon for 5 min. Then, naphthopyran **1** (70.8 mg, 0.14 mmol, 1.00 equiv) and 3 mL of degassed NMP were added. **CAUTION!**[[28]](#footnote-29) The resulting suspension was magnetically stirred for 44 h at 120 °C. After TLC monitoring indicated a complete consumption of the naphthopyran **1**, the reaction mixture was diluted with 5 mL of ethyl acetate and 5 mL of brine. After separation of the organic layer, the aqueous layer was extracted with ethyl acetate (5 × 5 mL). The combined organic layers were once washed with 5 mL of water, dried (MgSO_4_) and concentrated in vacuo. The crude product was purified by flash-chromatography (silica, pentane/diethyl ether, 6/1) affording furan **14** as a brown oil (39 mg, 9.1 × 10^−2^ mmol, 65%). *R_f_* = 0.79 (pentane/diethyl ether, 1/4). *t*_R_ = 10.2 min (flow rate: 1 mL/min, rt; eluent acetonitrile/water, gradient: isocratic for 20 min at 17/3 then within 10 min to pure acetonitrile). ^1^H NMR (400 MHz, CDCl_3_): [[29]](#footnote-30) *δ* = 8.69 (d, *J* = 1.5 Hz, 1 H), 8.13 (dd, *J* = 1.6 Hz, *J* = 8.6 Hz, 1 H), 8.05 (d, *J* = 8.6 Hz, 1 H), 7.80 (d, *J* = 8.9 Hz, 1 H), 7.65 (d, *J* = 8.9 Hz, 1 H), 7.23 – 7.19 (m, 4 H), 7.07 – 7.03 (m, 4 H), 6.75 (s, 1 H), 5.66 (s, 1 H), 3.99 (s, 3 H). ^13^C NMR (101 MHz, CDCl_3_):[[30]](#footnote-31) *δ* = 167.5 (Cq), 162.1 (d, *J*(F-C) = 246 Hz, Cq), 159.5 (Cq), 153.9 (Cq), 136.7 (d, *J*(F-C) = 3 Hz, Cq), 131.9 (CH), 130.5 (d, *J*(F-C) = 8 Hz, CH), 130.1 (Cq), 129.5 (Cq), 126.33 (CH), 126.29 (Cq), 126.0 (CH), 123.8 (CH), 123.5 (Cq), 115.8 (d, *J*(F-C) = 22 Hz, CH), 113.4 (CH), 105.0 (CH), 52.4 (CH_3_), 50.0 (CH). IR: (*ν*) = 3070 (w), 3041 (w), 2951 (w), 2929 (w), 2853 (w), 1717 (s), 1628 (w), 1603 (w), 1507 (s), 1401 (w), 1296 (m), 1261 (s), 1225 (s), 1196 (m), 1158 (m), 992 (w), 800 (m). MS (200 °C): *m/z* (%) = 428 (66) [M]^+^, 369 (12), 333 (100), 305 (36), 273 (36), 244 (13), 227 (36). HRMS calcd for C_27_H_18_F_2_O_3_, *m/z*: 428.1224; found: 428.1228.

3,3-Bis(4-fluorophenyl)-8-(methoxycarbonyl)-2-[(trimethylsilyl)ethinyl]-

3*H*-naphtho[2,1-*b*]pyran (**4**)

A Schlenk flask was charged with naphthopyran **1** (70 mg, 1.4 × 10^−1^ mmol, 1.0 equiv), Pd(PPh_3_)_4_ (16 mg, 1.4 × 10^−2^ mmol, 10 mol%) and CuI (4 mg, 2.1 × 10^−2^ mmol, 15 mol%). Then, THF (1 mL, 0.1 m in pyran **1**) and iPr_2_NH (39 *μ*L, 2.8 × 10^−1^ mmol, 2.0 equiv) were added. The yellow suspension was degassed and (trimethyl­silyl)­acetylene (39 *μ*L, 4.0 × 10^−1^ mmol, 2.8 equiv) was added. The mixture was magnetically stirred for 22 h at rt and the RP-HPLC monitoring indicated incomplete conversion. After additional stirring for 23 h at rt, RP-HPLC monitoring indicated a constant incomplete conversion and the solution was diluted with 5 mL of diethyl ether and 5 mL of brine. The layers were separated and the aqueous layer was extracted with diethyl ether (5 × 5 mL). The combined organic layers were washed with 5 mL of water, dried (MgSO_4_) and concentrated in vacuo. The remainder was purified by flash-chromatography (silica, pentane/diethyl ether, 10/1) to give 39 mg (7.4 × 10^−2^ mmol, 53% yield) of naphthopyran **4** as a brown solid. mp: 86 – 88 °C. *R_f_* = 0.59 (pentane/diethyl ether, 3/1). *t*_R_ = 14.0 min (flow rate: 1 mL/min, rt; eluent: acetonitrile/water, gradient: within 10 min from 17/3 to pure acetonitrile). UV-vis (acetonitrile, 2.5 × 10^−5^ m) *λ*_max_ (A): 369 nm (*ε* = 14488 dm^3^ × m^−1^ × cm^−1^), *λ*_max_ (B): 272 nm (*ε* = 46144 dm^3^ × m^−1^ × cm^−1^). ^1^H NMR (400 MHz, CDCl_3_): *δ* = 8.46 (d, *J* = 1.4 Hz, 1 H), 8.06 (dd, *J* = 8.8 Hz, *J* = 1.8 Hz, 1 H), 7.97 (d, *J* = 8.9 Hz, 1 H), 7.77 (d, *J* = 8.8 Hz, 1 H), 7.68 (s, 1 H), 7.48 – 7.45 (m, 4 H), 7.20 (d, *J* = 8.8 Hz, 1 H), 7.02 – 6.97 (m, 4 H), 3.96 (s, 3 H), 0.04 (s, 9 H). ^13^C NMR (101 MHz, CDCl_3_):[[31]](#footnote-32) *δ* = 167.2 (Cq), 162.6 (d, *J*(F-C) = 247 Hz, Cq), 152.5 (Cq), 138.5 (d, *J*(F-C) = 34 Hz, Cq), 132.5 (CH), 131.9 (Cq), 131.8 (CH), 130.2 (d, *J*(F-C) = 8 Hz, CH), 128.8 (Cq), 126.80 (CH), 126.75 (CH), 126.0 (Cq), 121.8 (CH), 120.8 (CH), 119.1 (CH), 115.0 (Cq), 114.8 (d, *J*(F-C) = 22 Hz, CH), 104.0 (Cq), 102.2 (Cq), 84.8 (Cq), 52.3 (CH_3_), –0.3 (CH_3_). IR: (*ν*) = 3072 (w), 2956 (m), 2899 (w), 2850 (w), 2138 (m) [C≡C], 1721 (s) [C=O], 1625 (m), 1603 (m), 1508 (s), 1467 (m), 1397 (w), 1280 (s), 1227 (s), 1202 (s), 1160 (s), 1087 (m), 857 (s). MS (RT °C): *m/z* (%) = 524 (100) [M]^+^, 509 (65), 451 (56), 432 (35), 430 (13), 429 (38), 392 (26). HRMS calcd for C_32_H_26_F_2_O_3_Si, *m/z*: 524.1619; found: 524.1622.

2,2-Bis(4-fluorophenyl)-6-({[(9*H*-fluoren-9-yl)methoxycarbonyl]amino}methyl)-2*H*‑1‑benzopyran-3-carboxylic acid **(6**)

Benzopyran **2b** (1.5 g, 2.30 mmol, 1.0 equiv), PdCl_2_(PPh_3_)_2_ (160 mg, 0.23 mmol, 10.0 mol%) and molecular sieve (115 mg, 4 Å, activated powder, 50 mg/0.1 mmol benzopyran)[[32]](#footnote-33) were mechanically suspended in 20 mL of degassed THF (0.1 m in benzopyran **2b**) in a 100-mL-steele-autoclave (Parr Instrument). The yellow suspension was stirred at rt for 30 min and degassed water (420 *μ*L, 23.0 mmol, 10.0 equiv) was added in a single portion. **CAUTION!**[[33]](#footnote-34) The suspension was mechanically stirred at 100 °C under a CO pressure of 50 atm for 72 h, and then concentrated in vacuo to give a black resin which was purified by flash-chromatography (silica, pentane/acetone, 7/4). Benzopyran **6** was isolated as an amorphous pale yellow wax (200 mg, 0.32 mmol, 14%). *R_f_ =* 0.30 (acetone/pentane, 2/3). Analytical RP-HPLC: *t*_R_ = 22.2 min (flow rate: 1 mL/min, rt; eluent: acetonitrile/water, gradient: within 25 min from 3/7 to 6/4). UV-vis (2.5 × 10^−5^ m, acetonitrile) *λ*_max_ (A): 339 nm (*ε* = 3256 dm^3^ × m^−1^ × cm^−1^), *λ*_max_ (B): 300 nm (*ε* = 10540 dm^3^ × m^−1^ × cm^−1^), *λ*_max_ (C): 265 nm (*ε* = 18584 dm^3^ × m^−1^ × cm^−1^). ^1^H NMR (400 MHz, CDCl_3_):[[34]](#footnote-35),[[35]](#footnote-36) *δ* = 7.77 – 7.75 (m, 3 H), 7.56 (d, *J* = 7.1 Hz, 2 H), 7.39 (t, *J* = 7.5 Hz, 2 H), 7.35 – 7.33 (m, 4 H), 7.31 – 7.27 (m, 2 H), 7.12 (d, *J* = 8.3 Hz, 1 H), 7.04 (s, 1 H), 6.97 – 6.92 (m, 4 H), 6.82 (d, *J* = 8.8 Hz, 1 H), 5.88 (br s, 1 H), 5.03 (br s, 1 H), 4.45 (d, *J =*6.7 Hz, 2 H), 4.23 – 4.18 (m, 3 H). ^13^C NMR (100.6 MHz, CDCl_3_): *δ* = 169.1 (Cq), 162.3 (d, *J*(F‑C) = 247 Hz, Cq), 156.6 (Cq), 152.9 (Cq), 143.9 (Cq), 141.5 (Cq), 138.3 (d, *J*(F-C) = 3 Hz, Cq), 136.6 (CH), 132.44 (CH), 132.38 (Cq), 130.5 (d, *J*(F-C) = 8 Hz, CH), 128.4 (Cq), 128.0 (CH), 127.9 (CH), 127.2 (CH), 125.1 (CH), 120.5 (Cq), 120.2 (CH), 117.4 (CH), 114.7 (d, *J*(F-C) = 22 Hz, CH), 84.6 (Cq), 66.8 (CH_2_), 47.4 (CH), 44.4 (CH_2_). IR: (*ν*) = 3335 (br, w), 3065 (w), 2957 (w), 2925 (w), 2854 (w), 1700 (s), 1601 (w), 1507 (s), 1378 (m), 1229 (s), 1159 (m), 992 (m), 840 (m). MS (240 °C): *m/z* (%) = 571 (<1) [M–CO_2_]^+^, 476 (3), 437 (3), 393 (9), 355 (12), 284 (65), 231 (12), 178 (62), 165 (12), 128 (100). HRMS: calcd for C_38_H_27_F_2_NO_5_: 615.1857; found: 615.1867.

**Spectroscopic data**

**UV-vis spectra** (2.5 × 10^−5^ m, acetonitrile):

**NMR spectra**

Discusson of impurties see footnote.[[36]](#footnote-37)

**COSY**:

HMQC:

HMBC:

**ORTEP plots:**


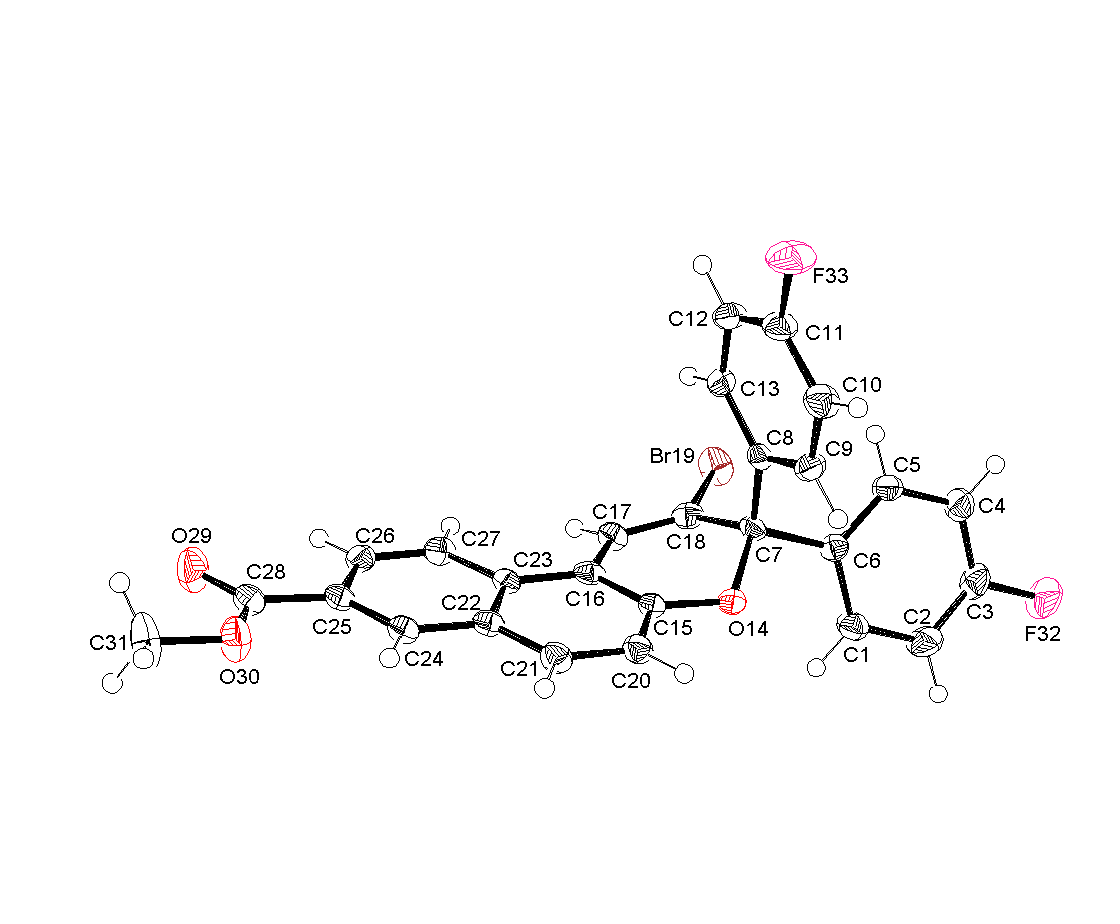


Figure S 2. ORTEP plot of compound **1**.


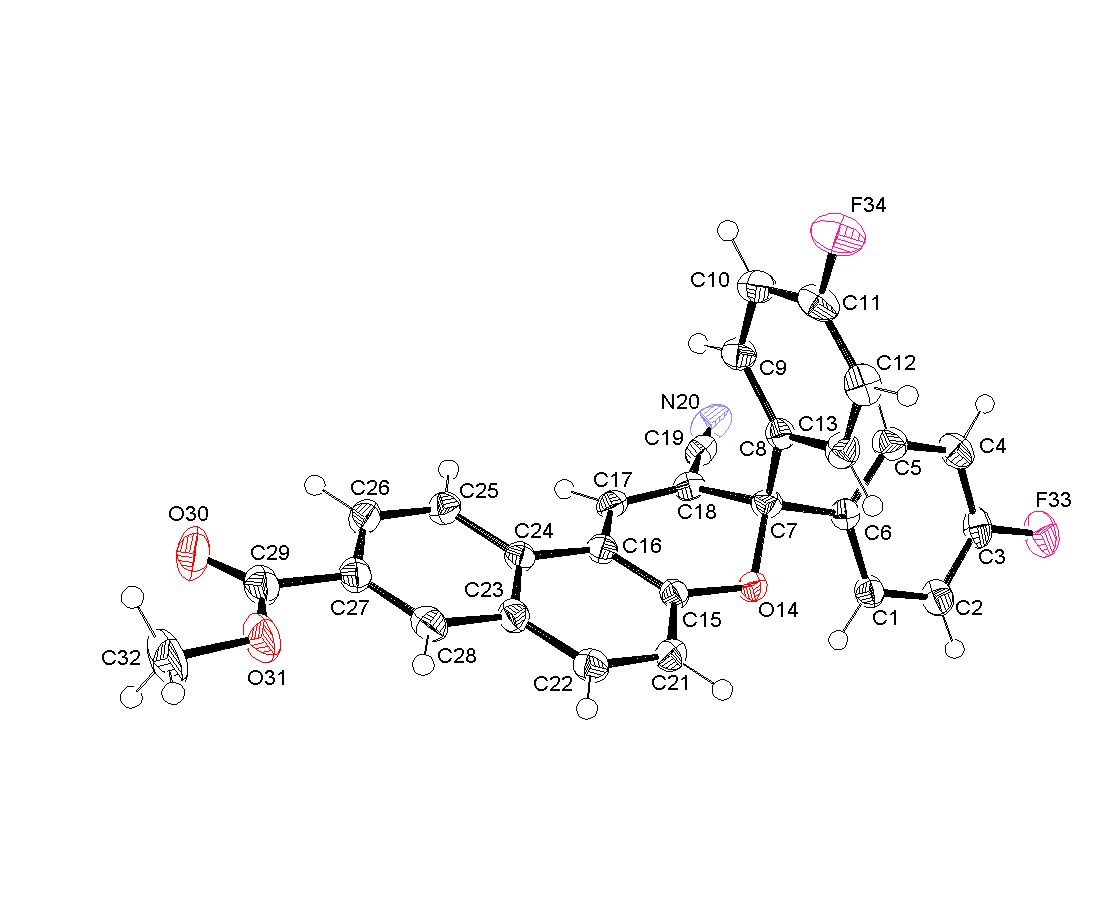


Figure S 3. ORTEP plot of compound **3**.


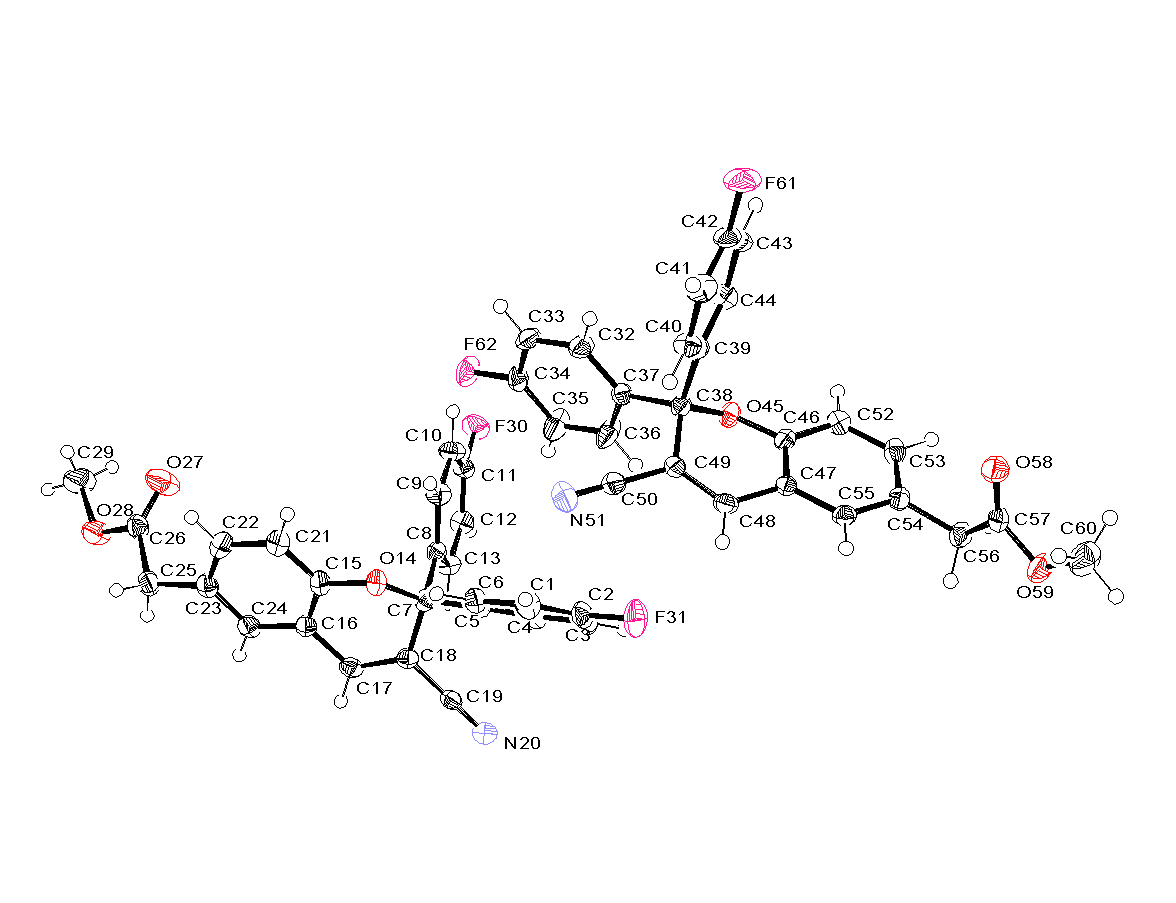


Figure S 1. ORTEP plot of compound **5a**.

References:

[1] Perrin, D. D.; Armarego, W. L. F. *Purification of Laboratorial Chemicals;* Wiley-VCH: Weinheim, 1998.

[2] Moss, G. P. *Pure Appl. Chem.* **1998,** *70,* 143–216. [doi:10.1351/pac199870010143](http://dx.doi.org/10.1351/pac199870010143)

[3] Claisen, L.; Eisleb, O. *Justus Liebigs Ann. Chem.* **1913,** *401,* 21–119.

[4] Partridge, M. W. *J. Chem. Soc.* **1949,** 3043–3046.

[5] Leuck, M.; Kunz, H. *Carbohydr. Res.* **1998,** *312,* 33–44. [doi:10.1016/S0008-6215(98)00223-7](http://dx.doi.org/10.1016/S0008-6215(98)00223-7)

[6] Penke, B.; Rivier, J. *J. Org. Chem.* **1987,** *52,* 1197–1200. [doi:10.1021/jo00383a004](http://dx.doi.org/10.1021/jo00383a004)

[7] Guibe, F.; Saint M'Leux, Y. *Tetrahedron Lett.* **1981,** *22,* 3591–3594. [doi:10.1016/S0040-4039(01)81967-5](http://dx.doi.org/10.1016/S0040-4039(01)81967-5)

[8] Roush, W. R.; Hartz, R. A.; Gustin, D. J. *J. Am. Chem. Soc.* **1999,** *121,* 1990–1991. [doi:10.1021/ja984229e](http://dx.doi.org/10.1021/ja984229e)

[9] Zhao, W.; Carreira, E. M. *Org. Lett.* **2003,** *5,* 4153–4154. [doi:10.1021/ol035599x](http://dx.doi.org/10.1021/ol035599x)

[10] Gabbutt, C. D.; Hepworth, J. D.; Heron, B. M.; Rahman, M. M. *J. Chem. Soc., Perkin Trans. 1* **1994,** 1733–1737. [doi:10.1039/P19940001733](http://dx.doi.org/10.1039/P19940001733)

[11] Schareina, T.; Zapf, A.; Beller, M. *Chem. Commun.* **2004,** 1388–1389. [doi:10.1039/b400562g](http://dx.doi.org/10.1039/b400562g)

**Part II Time-resolved photophysical spectroscopy**

**Material and Methods**

**Time-Resolved-Spectroscopy**

The chromene was dissolved in acetonitrile (as received from Merck, purity 99%) at a concentration in the millimolar range giving rise to an absorption of 0.4 OD at ~345 nm (optical path of 0.5 mm in a fused silica flow-cell). This stock solution was directly used for the time-resolved fs-experiments and diluted (1/10) for laser-flash-spectroscopy.

*Femtosecond pump-probe spectroscopy* in the visible and near ultraviolet spectral range was used to investigate the reaction dynamics of the photoinduced ring-opening of chromenes. The apparatus is described in more detail in ref. [1]. Briefly, a homebuilt Ti:Sa based laser-amplifier-system generated ultrashort pulses with a duration of ~100 fs at a repetition rate of ~1 kHz. The excitation pulses at 340 nm with an energy between 250 to 500 nJ where generated via a non-collinear optical parametric amplifier (NOPA) [2] in combination with frequency doubling in a 100 *μ*m BBO-crystal (type I). A white light continuum from CaF_2_ served as probe pulse (380 to 650 nm) [3] to record the absorption changes induced by the pump-pulse. The transient spectra at various delay times between pump and probe pulse were recorded using a multi-channel detection setup [4]. The instrumental response time (FWHM of the cross-correlation trace) was determined to be ~250 fs. The spot diameter of the pump-light was adjusted to ~120 *μ*m providing homogeneous excitation density for the probe-pulse (diameter ~60 *μ*m). The polarization of pump and probe pulses at the sample location were set magic angle (54.7°).

*A homebuilt laser-flash spectrometer* was used for the investigation of the transient absorbance changes in a time-range from nanoseconds to microseconds. The sample was excited with a pulsed Nd:YAG laser (3^rd^ harmonic, 355 nm, pulse duration ~7 ns) at a repetition rate of 40 Hz. The transient absorbance changes of the sample were investigated at ~460 nm using a high-power laser-LED (Luxeon, High Power LED, 1 W, Osram GmbH, 457 nm, ~20 nm spectral width) and a photodiode (Thorlabs DET10A/M) coupled to an oscilloscope (Tektronix TCS 320D). A temporal resolution of the setup of ~ 8 ns could be achieved. Further details are found in refs. [5,6].

# References

[1] Cordes, T.; Weinrich, D.; Kempa, S.; Riesselmann, K.; Herre, S.; Hoppmann, C.; Rück-Braun, K.; Zinth, W. *Chem. Phys. Lett.* **2006,** *428,* 167–173. [doi:10.1016/j.cplett.2006.07.043](http://dx.doi.org/10.1016/j.cplett.2006.07.043)

[2] Riedle, E.; Beutter, M.; Lochbrunner, S.; Piel, J.; Schenkl, S.; Spörlein, S.; Zinth, W. *Appl. Phys. B* **2000,** *71,* 457–465. [doi:10.1007/s003400000351](http://dx.doi.org/10.1007/s003400000351)

[3] Huber, R.; Satzger, H.; Zinth, W.; Wachtveitl, J. *Opt. Commun.* **2001,** *194,* 443–448. [doi:10.1016/S0030-4018(01)01324-4](http://dx.doi.org/10.1016/S0030-4018(01)01324-4)

[4] Seel, M.; Wildermuth, E.; Zinth, W. *Meas. Sci. Technol.* **1997,** *8,* 449–452. [doi:10.1088/0957-0233/8/4/014](http://dx.doi.org/10.1088/0957-0233/8/4/014)

[5] Schmidhammer, U.; Roth, S.; Riedle, E.; Tishkov, A. A.; Mayr, H. *Rev. Sci. Instrum.* **2005,** *76,* 093111. [doi:10.1063/1.2047828](http://dx.doi.org/10.1063/1.2047828)

[6] Elsner, C. Ultrakurzzeitspektroskopie zum Schaltverhalten substituierter Diarylethene. Ph.D. Thesis, LMU München, Germany, 2008.

1. Known compound. [↑](#footnote-ref-2)
2. The corresponding cif-files have been deposited at the Cambridge Crystallographic Data Centre (CCDC), 12, Union Road, Cambridge CB2 1EZ, UK, and copies can be obtained on request, free of charge, by quoting the publication citation and the deposition numbers 688509 (**1**), 688510 (**3**) and 688508 (**5a**). These data can also be obtained free of charge via www.ccdc.cam.ac.uk/data_request/cif, by emailing data_request@ccdc.cam.ac.uk, or by contacting fax: +44 1223 336033. [↑](#footnote-ref-3)
3. SCALE3 ABSPACK and CrysAlisRed (version 1.171.29.10), Oxford Diffraction Ltd, Abingdon, Oxford, England, 2006. [↑](#footnote-ref-4)
4. G. M. Sheldrick, SHELX97, *Program package for the solution and refinement of crystal structures*, Release 97-2, University of Göttingen, Germany, 1997. [↑](#footnote-ref-5)
5. The broad signals at *δ* = 7.14 – 7.02 and 5.03 ppm were assigned to the carbamate-N-H. [↑](#footnote-ref-6)
6. Due to the symmetry of the carbamate, its ^13^C NMR-spectrum contains *eight* signals *less* (i.e. a total of C*_17_*) than there are carbon atoms from elemental analysis (C*_25_*H_23_NO_3_). [↑](#footnote-ref-7)
7. The broad signals at *δ* = 7.07 – 6.91 and 5.02 ppm were assigned to the carbamate-N-H. [↑](#footnote-ref-8)
8. Due to the symmetry of carbamate **8b**, its ^13^C NMR-spectrum contains *eight* signals *less* (i.e. a total of C*_14_*) than there are carbon atoms from elemental analysis (C*_22_*H_19_NO_3_). [↑](#footnote-ref-9)
9. Due to the symmetry of naphthopyran **10**, its ^13^C NMR-spectrum contains *eight* signals *less*
   (i.e. a total of C*_19_*) than there are carbon atoms from elemental analysis (C*_27_*H_18_F_2_O_3_). [↑](#footnote-ref-10)
10. The signal at 139.7 ppm, corresponding to C-4 (see figure above), is not resolved and thus doesn’t show the coupling constant of ^4^*J*(F-C) = 3 Hz, which is found in most of the similar compounds (e.g. naphthopyran **10** at 140.3 ppm). Moreover, due to the symmetry of bromohydrin **11**, its ^13^C NMR-spectrum contains *four* signals *less* (i.e. a total of C*_23_*) than there are carbon atoms from elemental analysis (C*_27_*H_19_BrF_2_O_4_). [↑](#footnote-ref-11)
11. Due to the symmetry of naphthopyran **1**, its ^13^C NMR-spectrum contains *eight* signals *less* (i.e. a total of C*_19_*) than there are carbon atoms from elemental analysis (C*_27_*H_17_BrF_2_O_3_). [↑](#footnote-ref-12)
12. Due to the symmetry of benzopyran **12a**, its ^13^C NMR-spectrum contains *eight* signals *less* (i.e. a total of C*_16_*) than there are carbon atoms from elemental analysis (C*_24_*H_18_F_2_O_3_). [↑](#footnote-ref-13)
13. The broad signals at *δ* = 4.17, 5.01, 7.25 and 7.50 were assigned to the carbamate-N-H. [↑](#footnote-ref-14)
14. Due to the symmetry of benzopyran **12b**, its ^13^C NMR-spectrum contains *fourteen* signals *less* (i.e. a total of C*_23_*) than there are carbon atoms from elemental analysis (C*_37_*H_27_F_2_NO_3_). [↑](#footnote-ref-15)
15. Due to the symmetry of bromohydrin **13a**, its ^13^C NMR-spectrum contains *four* signals *less*(i.e. a total of C*_20_*) than there are carbon atoms from elemental analysis (C*_24_*H_19_BrF_2_O_4_). [↑](#footnote-ref-16)
16. Due to the symmetry of bromohydrin **13b**, its ^13^C NMR-spectrum contains *ten* signals *less* (i.e. a total of C*_27_*) than there are carbon atoms from elemental analysis (C*_37_*H_28_BrF_2_NO_4_). [↑](#footnote-ref-17)
17. Due to the symmetry of benzopyran **2a**, its ^13^C NMR-spectrum contains *eight* signals *less* (i.e. a total of C*_16_*) than there are carbon atoms from elemental analysis (C*_24_*H_17_BrF_2_O_3_). [↑](#footnote-ref-18)
18. The broad signals at *δ* = 4.17 and 4.97 were assigned to the carbamate-N-H. [↑](#footnote-ref-19)
19. Due to the symmetry of benzopyran **2b**, its ^13^C NMR-spectrum contains *fourteen* signals *less* (i.e. a total of C*_23_*) than there are carbon atoms from elemental analysis (C*_37_*H_26_BrF_2_NO_3_). [↑](#footnote-ref-20)
20. **CAUTION!** Cyanide and gaseous HCN are highly toxic and need special precautions. For additional information see: international chemical safety cards (ICSCs) at www.ilo.org or www.hvbg.de/e/bia/gestis/. [↑](#footnote-ref-21)
21. Due to the symmetry of naphthopyran **3**, its ^13^C NMR-spectrum contains *eight* signals *less* (i.e. a total of C*_20_*) than there are carbon atoms from elemental analysis (C*_28_*H_17_F_2_NO_3_). [↑](#footnote-ref-22)
22. **CAUTION!** Cyanide and gaseous HCN are highly toxic and need special precautions. For additional information see: international chemical safety cards (ICSCs) at www.ilo.org or www.hvbg.de/e/bia/gestis/. [↑](#footnote-ref-23)
23. Due to the symmetry of benzopyran **5a**, the ^13^C NMR-spectrum contains *eight* signals *less* (i.e. a total of C*_17_*) than there are carbon atoms from elemental analysis (C*_25_*H_17_F_2_N_1_O_3_). [↑](#footnote-ref-24)
24. **CAUTION!** Cyanide and gaseous HCN are highly toxic and need special precautions. For additional information see: international chemical safety cards (ICSCs) at www.ilo.org or www.hvbg.de/e/bia/gestis/. [↑](#footnote-ref-25)
25. The broad signal at *δ* = 5.03 was assigned to the carbamate-N-H. [↑](#footnote-ref-26)
26. Impurities and residual solvents can be removed by RP-HPLC. [↑](#footnote-ref-27)
27. Due to the symmetry of benzopyran **5b**, its ^13^C NMR-spectrum contains *fourteen* signals *less* (i.e. a total of C*_24_*) than there are carbon atoms from elemental analysis (C*_38_*H_26_F_2_N_2_O_3_). [↑](#footnote-ref-28)
28. **CAUTION!** Experiments in pressure tubes at high temperatures are potentially hazardous and must only be carried out by using appropriate equipment and safety precautions. A blast shield should be in place and vessels designed to withhold elevated pressures must be used. After completion of an experiment, the vessel must be allowed to cool before opening to the atmosphere. [↑](#footnote-ref-29)
29. Impurities and residual solvents can be removed by RP-HPLC. [↑](#footnote-ref-30)
30. Due to the symmetry of napthofuran **14**, its ^13^C NMR-spectrum contains *eight* signals *less* (i.e. a total of C*_19_*) than there are carbon atoms from elemental analysis (C*_27_*H_18_F_2_O_3_). [↑](#footnote-ref-31)
31. Due to the symmetry of naphthopyran **4**, its ^13^C NMR-spectrum contains *ten* signals *less* (i.e. a total of C*_22_*) than there are carbon atoms from elemental analysis (C*_32_*H_26_F_2_O_3_Si). [↑](#footnote-ref-32)
32. The powder was dried prior to use (~750 °C, <1 mbar). [↑](#footnote-ref-33)
33. **CAUTION!** CO is highly toxic and needs special precautions. For additional information see: international chemical safety cards (ICSCs) at www.ilo.org or www.hvbg.de/e/bia/gestis/. [↑](#footnote-ref-34)
34. The broad signal at *δ* = 5.03 was assigned to the carbamate-N-H. [↑](#footnote-ref-35)
35. Impurities and residual solvents can be removed by RP-HPLC. [↑](#footnote-ref-36)
36. The impurities below 2.5 ppm didn’t give any detectable ^1^H-^1^H-correlation (see page S 58 for COSY spectra) and no detectable ^1^H-^13^C-correlation to the ^13^C-NMR-signals of compound **5b** (see page S 59 – S 60 for the corresponding HMBC and HMQC spectra). [↑](#footnote-ref-37)
